# Exact Probability Distributions of Selected Species in Stochastic Chemical Reaction Networks

**DOI:** 10.1007/s11538-014-9985-z

**Published:** 2014-08-26

**Authors:** Fernando López-Caamal, Tatiana T. Marquez-Lago

**Affiliations:** Integrative Systems Biology Unit, Okinawa Institute of Science and Technology, Kunigami, Okinawa 904-0412 Japan

**Keywords:** Chemical master equation, Analytical solutions, Model reduction

## Abstract

Chemical reactions are discrete, stochastic events. As such, the species’ molecular numbers can be described by an associated master equation. However, handling such an equation may become difficult due to the large size of reaction networks. A commonly used approach to forecast the behaviour of reaction networks is to perform computational simulations of such systems and analyse their outcome statistically. This approach, however, might require high computational costs to provide accurate results. In this paper we opt for an analytical approach to obtain the time-dependent solution of the Chemical Master Equation for selected species in a general reaction network. When the reaction networks are composed exclusively of zeroth and first-order reactions, this analytical approach significantly alleviates the computational burden required by simulation-based methods. By building upon these analytical solutions, we analyse a general monomolecular reaction network with an arbitrary number of species to obtain the exact marginal probability distribution for selected species. Additionally, we study two particular topologies of monomolecular reaction networks, namely (i) an unbranched chain of monomolecular reactions with and without synthesis and degradation reactions and (ii) a circular chain of monomolecular reactions. We illustrate our methodology and alternative ways to use it for non-linear systems by analysing a protein autoactivation mechanism. Later, we compare the computational load required for the implementation of our results and a pure computational approach to analyse an unbranched chain of monomolecular reactions. Finally, we study calcium ions gates in the sarco/endoplasmic reticulum mediated by ryanodine receptors.

**Electronic supplementary material:**

The online version of this article (doi:10.1007/s11538-014-9985-z) contains supplementary material, which is available to authorized users.

## Introduction

Chemical reactions are random events. Although this might suggest an uncontrolled behaviour, difficult to analyse or predict, they are the basis of highly organised phenomena happening in living systems. Despite this relevance, there are still many gaps in understanding the principles governing the regulatory systems that deal with such randomness. Thus, a means for forecasting the behaviour of stochastic reaction networks in an efficient, yet accurate manner is essential to understand cellular regulatory mechanisms.

Stochastic systems can reproduce chemical reaction dynamics (Gardiner et al. 1985; Iglesias and Ingalls 2010). However, the large number of reactions in the network and their nonlinear nature hinder an analytical treatment of the corresponding stochastic models. To overcome this difficulty, it is common practice to handle such models numerically. The Stochastic Simulation Algorithm, SSA, (Gillespie 1977; Kurtz 1972) is a widely used computational approach to obtain time courses of species’ molecular numbers. We refer the interested reader to Gillespie (2007), Barrio et al. (2010) and Erban et al. (2007) for a survey of simulation methods for stochastic reaction networks. By performing a statistical analysis on the trajectories obtained by simulation, one can identify properties of the studied reaction network. One of the most insightful properties is the time-dependent probability density function that describes the probability of having a specific number of molecules for each species. However, the number of simulations required for this computational approach might become prohibitively large due to the size, type of reactions, and kinetic parameters of the reaction network.

The chemical master equation (CME) is a set of ordinary differential equations associated to a continuous-time, discrete-state Markov Chain. This Markov Chain describes the temporal evolution of the probability density function of having certain species’ molecular counts. Although an analytical treatment of the CME is in general challenging, there are classes of reaction networks for which closed-form analytical solutions are available. Some members of these classes are reaction networks that have only one reactant per reaction, denoted as monomolecular or first-order reaction networks. Jahnke and Huisinga (2007) derived an analytical solution for the CME for such systems, whereas Gadgil et al. (2005) obtained expressions for the first two moments of the probability density function arising from associated CMEs. In turn, Lee and Kim (2012) obtained the analytical solution for the CME of classes of nonlinear reactions, by casting the state transition as a Markov Chain that resembles that of a monomolecular reaction network.

In this paper, we obtain closed-form expressions for the time-dependent solution of the CME associated to general stochastic reaction networks. We note that, as the numbers of molecules and reactions increase, the order of the CME explodes; thereby, hindering the applicability of this analytical treatment. Later, by availing of the results in Jahnke and Huisinga (2007), we obtain an exact analytical solution of the CME for selected species in an arbitrarily large monomolecular reaction network. For these monomolecular reaction networks, the order of the ODEs to solve is those of the number of species in the reaction network, in contrast to the number of states of the CME in a general reaction network. In addition to considering general monomolecular reaction networks, we focus on two different topologies with an arbitrary number of species: (i) an unbranched chain of monomolecular reactions with and without synthesis and degradation of the species and (ii) a chain of unbranched monomolecular reactions in which the last species also interacts with the first species, therefore, creating a ring of reactions.

It should be noted that Leier et al. (2014) and Barrio et al. (2013) tackled the problem of exactly reducing general monomolecular reaction networks and unbranched monomolecular reaction networks, respectively, into one monomolecular reaction. This reduced representation is characterised by a constant reaction rate and a time delay sampled from a defined distribution. In addition to obtaining a simpler representation, the authors achieved savings on the computational load required for the simulation of the reduced model. However, we note that such approaches use the delay stochastic simulation algorithm (DSSA, see Cai 2007; Barrio et al. 2006, 2010, for instance). In contrast, our methodology obtains an exact, analytical solution of the CME of the reaction network studied in Barrio et al. (2013) and additional topologies, hence avoiding the computational burden of the (D)SSA.

To exemplify the applicability of our results, we analyse a protein autoactivation mechanism with nonlinear reaction propensities. Later, we compare the computational time required to obtain the solution of the CME of an unbranched, monomolecular reaction network via our methodology and the implementation of four simulation algorithms: (i) the Stochastic Simulation Algorithm, SSA, (Gillespie 1977; Kurtz 1972) (ii) the Next Reaction Method, NRM, (Gibson and Bruck 2000), (iii) Optimized Direct Method, ODM (Cao et al. 2004), and (iv) a hybrid stochastic simulation algorithm (Liu et al. 2012). In such an example, we note that the computational time required by our approach is similar to a few runs of the SSA. To conclude, we also analyse a ring of monomolecular reactions inspired by a model of ion gating mediated by ryanodine receptors (Lanner et al. 2010; Keener 2009).

## Chemical Reaction Networks

A group of $n$ species $S_i$ interacting via $m$ reactions may be represented by

1 $$\begin{aligned} \sum _{i=1}^n \alpha _{ij} S_i \mathop {\rightleftharpoons }\limits _{}^{v_j} \sum _{i=1}^n \beta _{ij} S_i . \end{aligned}$$

Here $v_j$ denotes the rate of the $j$th reaction and $\alpha _{ij} \text { and } \beta _{ij}$ are known as the stoichiometric coefficients. In matrix form, a mathematical model of (1) is

2 $$\begin{aligned} \frac{\mathrm {d}}{\mathrm {d} t }\mathbf {{c}}( t ) = \mathbf {Nv({c}( t ))}, \quad \mathbf {{c}}(0) = \mathbf {{c}}_0. \end{aligned}$$

Here $\mathbf {{c}( t )} : {\mathbb {R}}_+ \rightarrow {\mathbb {R}}_+^{n}$ is a column vector that contains the species’ concentration in (1). In turn, the $j$th entry of $\mathbf {v({c}( t ))} : {\mathbb {R}}_+\times {\mathbb {R}}_+^n \rightarrow {\mathbb {R}}^{m}$ is the reaction rate of the $j$th reaction, $v_j$. When a reaction has more than one reactant, the rate $v_j$ is a nonlinear function, which can be modelled by the Mass Action Law (Chellaboina et al. 2009), for instance. With the definitions above, the mass action reaction rate of the $j$th reaction in (1) has the form

$$\begin{aligned} v_j(\mathbf {c}( t ) ) = \delta _j \prod _{i=1}^{n} c_i^{\alpha _{ij}}( t ), \end{aligned}$$

where $\delta _j$ is a parameter that depends on the nature of the reaction taking place. Please, refer to Table 1 for the reaction rates of the most common reactions. The link between the reaction rates to the change of the species’ concentration is the stoichiometric matrix $\mathbf {N} \in \mathbb {N}^{n \times m}$, whose $ij$th entry is defined as

3 $$\begin{aligned} n_{ij} := \beta _{ij} - \alpha _{ij}. \end{aligned}$$

When using any approach based on a continuous representation of $\mathbf {{c}}( t )$, a large number of molecules within a well-stirred spatial domain are assumed. This assumption, however, is not appropriate for modelling all biochemical systems. In the following section, we present a stochastic formulation of the dynamics of the species’ molecular number in the reaction network (1).

### Stochastic Formulation

When the species’ molecular number in a reaction network is low, a continuous representation of molecules number fails to represent the actual behaviour of the reaction network. There are several reasons for this. First, for some biochemical processes, the number of reactants might only be a few molecules. In such cases, a fraction of a molecule is meaningless and a discrete description of the species’ molecular count is essential. Let us consider a set that contains all the possible combinations of number of molecules, $\mathbf {s}^i$, that the reaction network can exhibit:

4 $$\begin{aligned} \mathbf {S}:= \left\{ \mathbf {s}^{i} \in \mathbb Z^n, \, \forall \, i \in [1,w] \right\} . \end{aligned}$$

Here $\mathbf {s}( t ): {\mathbb {R}}_+ \rightarrow \mathbf {S}$ is a column vector whose $j$th entry represents the number of molecules, $s_j( t )$, of the species $S_j$. We note that (4) shows that the entries of $\mathbf {s}(\circ )$ are integer numbers, in contrast to the continuous, deterministic formulation.

Second, when we consider a low number of molecules in a reaction network, the reaction rates can no longer be modelled by the Law of Mass Action. This follows from the fact that the rate at which reactants become products does not depend only on the temperature and specific properties of the meeting reactants, but also on whether the reactants actually meet in a timely fashion. Let us assume that the $k$th reaction is the only reaction occurring in the time interval $[ t , t + \tau )$. Hence the number of molecules at time $t+\tau$ is

5 $$\begin{aligned} \mathbf {s}( t + \tau ) = \mathbf {s}( t ) + \mathbf {n}^{k}, \end{aligned}$$

where $\mathbf {n}^{k}$ represents the $k$th column of the stoichiometric matrix $\mathbf {N}$ in (2). In this framework, Gillespie (1977) considered a measure of the probability of the $k$th reaction to occur in the time interval $[ t , t + \mathrm {d} t )$, given that the number of molecules in time $t$ is $\mathbf {s}( t )$. This probability is given by $a_k(\mathbf {s}( t ))\mathrm {d} t$, where $a_k(\mathbf {s}( t ))$ is the so-called reaction propensity and represents the reaction probability per unit of time of the $k$th reaction. The propensities for some common reactions and their relationship with the corresponding velocities of reaction are given in Table 1.

**Table 1:** Different kinds of chemical reactions and their associated velocity and propensity

Reaction	Velocity	Propensity	Parameter relationship
$0 \xrightarrow {} S_1$	$v_1= \delta _1$	$a_1 = k_1$	$k_1 = {\mathcal {N}}{\mathcal {V}} \delta _1$
$$S_2 \xrightarrow {} X$$	$$v_2= \delta _2 c_2 ( t ) $$	$$a_2 = k_2 s_2( t ) $$	$$k_2 = \delta _2$$
$S_3 + S_4 \xrightarrow {} X$	$v_3= \delta _3 c_3 ( t )c_4( t )$	$a_3 = k_3 s_3( t ) s_4( t )$	$k_3 = \delta _3 / ({\mathcal {N}}{\mathcal {V}})$
$2S_5 \xrightarrow {} S_5 + X$	$v_4= \delta _4 c_5^2 ( t )$	$a_4 = k_4 s_5( t ) (s_5( t )-1) \slash 2$	$k_4 = 2\delta _4 / ({\mathcal {N}}{\mathcal {V}})$

The symbol $S_i$ denotes a species, $c_i$ represents the concentration of species $S_i$, and $v_j(\circ )$ is the velocity of the $j$th reaction. Likewise, $s_i$ denotes the number of $S_i$ molecules and $a_j(\circ )$ represents the propensity of the $j$th reaction. The units of concentrations are in molar $[\mathrm M]$, the volume $\mathcal {V}$ is in litres $[\mathrm l]$, and the Avogadro constant is ${\mathcal {N}} = 6.02214\times 10 ^ {23}~ [\mathrm {molecules}/\mathrm {mol}]$

By comparing the value of all the reactions propensities, one can determine which reaction is more likely to happen within the time-interval $[ t , t +\tau )$. This is the basic idea behind the SSA. For a thorough explanation of this algorithm and the overview of alternative simulation algorithms including $\tau$-leap methods, delays, and reaction–diffusion simulation algorithms, we refer the interested reader to Barrio et al. (2010); Cai (2007); Erban et al. (2007). Also, there exist recent formulations of exact stochastic simulation algorithms whose aim is to reduce some superfluous calculations in the SSA; for instance, the next reaction method, NRM (Gibson and Bruck 2000) and optimized direct method, ODM (Cao et al. 2004).

Single SSA runs yield trajectories that describe the species’ number of molecules in time. However, it is difficult to infer properties of the reaction network from single implementations of this algorithm. To obtain a better description of the system, a usual strategy is to run the SSA a large number of times and to extract statistical information from the outcome of these simulations. However, this approach might be computationally demanding and, hence, the study of intricate reaction networks might become infeasible.

An alternative way to discern some of the properties of the reaction network is to analyse an associated ODE. The solution of such an ODE describes the probability of the reaction network’s state to be $\mathbf {s}^i$ at time $t$, which we further denote as $p\left( \mathbf {s}^i, t \right)$. This ODE is known as the CME:

6 $$\begin{aligned} \frac{\mathrm {d}}{\mathrm {d} t }p\left( \mathbf {s}^i, t \right)&=- p\left( \mathbf {s}^i, t \right) \sum _{k=1}^{m} a_k(\mathbf {s}^i) + \sum _{k=1}^{m}p\left( \mathbf {s}^i - \mathbf {n}^k, t \right) a_k(\mathbf {s}^i-\mathbf {n}^k). \end{aligned}$$

Let

7 $$\begin{aligned} \mathbf {p}( t ) := \left( p\left( \mathbf {s}^1, t \right) \quad \ldots \quad p\left( \mathbf {s}^w, t \right) \right) ^T : \mathbf {S}\times {\mathbb {R}}_+ \rightarrow [0,1]^w, \end{aligned}$$

where $w$ was introduced in (4) as the number of distinct states $\mathbf {s}^i$ that the reaction network (1) can exhibit. Then, Equation (6) in matrix form is

8 $$\begin{aligned} \frac{\mathrm {d}}{\mathrm {d} t }\mathbf {p}( t ) = \mathcal {A} \mathbf {p}( t ) , \quad \mathbf {p}(0) = \mathbf {p}_0, \end{aligned}$$

where the entries of ${\mathcal {A}} \in {\mathbb {R}}^{w\times w}$ are given by

9 $$\begin{aligned} {\mathcal {A}}_{ji} = {\left\{ \begin{array}{ll} -\sum _{k=1}^{m} a_k(\mathbf {s}^i), &{} i=j,\\ a_k(\mathbf {s}^i), &{} \forall \, j: \mathbf {s}^j = \mathbf {s}^i + \mathbf n^k,\\ 0, &{} \text {otherwise}. \end{array}\right. } \end{aligned}$$

When the number of states $\mathbf {s}^i$ are finite, it is possible to obtain the closed form solution of the CME in (8). In turn, when $w$ is prohibitively large or infinite one may use the Finite State Projection method (Munsky and Khammash 2006) to obtain a matrix ${\mathcal {A}}$ with finite number of states, which leads to an approximation of the solution of the CME. In the following section, we present exact formulae for the solution of linear ODEs which can be used to derive closed-form analytical solutions of (9).

### Linear ODEs Analytical Solution

The forthcoming propositions present explicit formulae for the solution of finite-dimensional, time-invariant, linear ODEs of the following form

10a $$\begin{aligned} \frac{\mathrm {d}}{\mathrm {d} t }\mathbf {e}( t )&=\mathbf {A} \mathbf {e}( t ),\quad \mathbf {e}(0) = \mathbf {e}_0, \end{aligned}$$

10b $$\begin{aligned} \mathbf y( t )&=\mathbf {C}\mathbf {e}( t ), \end{aligned}$$

where $\mathbf {e}( t ):{\mathbb {R}}_+\rightarrow {\mathbb {R}}^n$, $\mathbf {A} \in {\mathbb {R}}^{n\times n}$, and $\mathbf C$ selects a defined number of states of $\mathbf {e}( t )$. The following proposition presents a formula for $\mathbf y ( t )$, when the eigenvalues of $\mathbf {A}$ are simple.

#### **Proposition 1**

Consider a linear system of the form (10), and assume that the eigenvalues, $\lambda _i\, \forall ~i \in [1,n]$, of $\mathbf {A}$ are simple. Then

11 $$\begin{aligned} \mathbf y( t ) = \sum _{\ell ={1}}^n \vartheta _{\ell }\exp (\lambda _{\ell } t)\mathbf {M} (\lambda _{\ell })\mathbf {e}_0, \end{aligned}$$

where $\lambda _{\ell }$ is the $\ell$th eigenvalue of $\mathbf {A}$, and $\mathbf {M}(\lambda _{\ell })$ is as follows

12a $$\begin{aligned} \mathbf {M} (\lambda _{\ell })&:= \mathbf C \begin{pmatrix} m_{11}(\lambda _{\ell }) &{}\quad m_{21}(\lambda _{\ell })&{}\quad \ldots &{}\quad m_{n1}(\lambda _{\ell })\\ \vdots &{}\quad \vdots &{}\quad \ddots &{}\quad \vdots \\ m_{1n}(\lambda _{\ell }) &{}\quad m_{2n}(\lambda _{\ell })&{}\quad \ldots &{}\quad m_{nn}(\lambda _{\ell }) \end{pmatrix}. \end{aligned}$$

Here $m_{ij}(\lambda _{\ell })$ is the $ij$th cofactor of the matrix $\lambda _{\ell } \mathbf {I_{ n }} - \mathbf {A}$. In addition,

12b $$\begin{aligned} \vartheta _{\ell }&:= \left( \prod _{k=1,k\ne \ell }^{n} \lambda _{\ell }-\lambda _k \right) ^{-1}. \end{aligned}$$

The proof can be found in Appendix. We note that the expression (11) can be rewritten in matrix form as

13 $$\begin{aligned} \mathbf {y}( t )= {\mathcal {D}} \exp (\varvec{\lambda } t ), \end{aligned}$$

where the column vector $\varvec{\lambda }\in {\mathbb {R}}^n$ contains all the eigenvalues of $\mathbf {A}$ in (10); in turn, $\exp (\varvec{\lambda } t )$ is the element-wise application of the exponential function to the vector $\varvec{\lambda } t$; and the $\ell$th column of ${\mathcal {D}}$ is

$$\begin{aligned} {\mathcal {D}}^\ell := \vartheta _{\ell } \mathbf {M}(\lambda _{\ell })\mathbf {e}_0. \end{aligned}$$

We note that the formula in (13) can be derived in terms of the exponential matrix: $\mathbf {y}( t ) = \mathbf {C}\exp ( \mathbf {A} t )\mathbf {e}_0$ (Gantmakher 1959, p. 118). However, calculating the exponential matrix is known to be a slow computational process. In turn, the formula in (13) relies on an accurate computation of the eigenvalues. The following corollary considers the case in which all the initial concentrations are zero except for that of the $i$th state.

#### **Corollary 1**

Consider the system analysed in Proposition 1. Additionally, let the $j$th entry of the initial condition vector be the only nonzero initial condition: $e_i(0) = 0\,\forall ~i\ne j$. Then

$$\begin{aligned} \mathbf y( t )&=e_j(0) \sum _{\ell ={1}}^n \vartheta _{\ell }\exp (\lambda _\ell t)\mathbf {m}^j(\lambda _\ell ), \end{aligned}$$

where $\mathbf {m}^j(\lambda _\ell )$ is the $j$th column of $\mathbf {M}(\lambda _\ell )$ defined in (12a).

The proof follows directly from evaluating (11) with the initial condition described above. In Proposition 1 we assumed that the eigenvalues of $\mathbf {A}$ are simple. Although unlikely, in some cases the eigenvalues might be repeated. We refer the interested reader to the Supplemental Online Material (SOM) for the case of repeated eigenvalues. Moreover, we note that the expressions derived in this section can be directly used to solve the CME in (8).

We note that ${\mathcal {A}}$ in (8) is a square matrix of order $w$, where $w$ is the number of states of the CME. This suggests that even for simple reaction topologies with tens or hundreds of molecules, the number of differential equations to simultaneously solve becomes prohibitively large. As the number of states increases, the dimension of the CME explodes, rendering infeasible the computation of eigenvalues $\lambda _\ell$ and cofactor matrices ${\mathbf {M}}(\lambda _\ell )$ required for the evaluation of the analytical solution. Alternatively, the numerical solution of the ODEs defining the CME might be able to handle larger systems than the analytical approach, at the cost of needing to assess stability and accuracy of such numerical approach.

In cases in which the number of states of the CME is prohibitively large or even infinite, it is possible to use the Finite State Projection method (Munsky and Khammash 2006) or the approach described in López-Caamal and Marquez-Lago (2014), for example. From these approaches one can obtain a matrix $\mathcal {A}$ with a smaller, finite number of states. Such a matrix can be used to obtain approximation of the CME via the analytical solutions described in this section.

In the following section, we analyse more specific reaction networks composed exclusively of zeroth and first-order reactions, for which exact analytical solutions can be obtained along with noticeable reduction of the computational burden. This follows from the fact that the ODEs required to derive such solutions will be of the order of the number species in the reaction network $(n)$, rather than the number of states of the CME $(w)$.

## Monomolecular Reaction Networks

In this section, we analyse reaction networks solely composed of the reaction networks shown in Table 2, and denote them in the following as monomolecular reactions. To overcome the shortcomings related to the computational burden, Jahnke and Huisinga (2007) derived the closed-form solution of the CME that models a monomolecular reaction network. In this section, we will focus on such reaction networks and we present the closed-form, analytical solution of the probability distributions for each state in such reaction networks.

**Table 2:** Types of monomolecular reactions and their propensities

Reaction	Propensity
$S_i \xrightarrow {k_{\mathrm {f} 1}} S_j$	$a_1 = k_{\mathrm {f} 1} s_i( t )$
$0 \xrightarrow {k_{\mathrm {s} 2}} S_\ell$	$a_2 = k_{\mathrm {s} 2}$
$S_m \xrightarrow {k_{\mathrm {d} 3}} 0$	$a_3 = k_{\mathrm {d} 3}s_m( t )$

### Analytical Solution of the CME of Monomolecular Reaction Networks

Let us consider the following reaction network

14 $$\begin{aligned} S_i \mathop {\rightleftharpoons }\limits _{k_{\mathrm {b} i}}^{k_{\mathrm {f} i}} S_{j}, \quad S_i \xrightarrow {k_{\mathrm {d} i}} 0, \quad 0 \xrightarrow {k_{\mathrm {s} i}} S_i, \quad \forall \, i,j \in [1,n],~i \ne j. \end{aligned}$$

The constants $k_{\mathrm {f} i}, k_{\mathrm {b} i}, k_{\mathrm {d} i}, k_{\mathrm {s} i}$ are nonnegative real numbers characterising the propensity reaction rates described in Table 2. For now, we will assume that not all the degradation constants are zero. The stoichiometric matrix $\mathbf N$ and the reaction rate vector $\mathbf {v}(\mathbf {c}( t ))$ in (2) are partitioned as follows

15a $$\begin{aligned} \mathbf {N}&=\begin{pmatrix} \mathbf {N_{\scriptscriptstyle {L}}}&\quad \mathbf {N_{0}}\end{pmatrix},\end{aligned}$$

15b $$\begin{aligned} \mathbf {v}(\mathbf {c}( t ))&=\begin{pmatrix} \left( \mathbf {G \mathbf {c}( t )}\right) ^T&\quad \mathbf {v_{0}}^T \end{pmatrix}^T. \end{aligned}$$

Here $\mathbf {N_{\scriptscriptstyle {L}}}$ gathers the stoichiometric vectors of the linear reactions and $\mathbf {N_{0}}$ those of the zeroth-order reactions (synthesis rates). The dimensions of the matrices above are $\mathbf {N_{\scriptscriptstyle {L}}}\in {\mathbb {R}}^{n \times m_{\scriptscriptstyle {L}}},\mathbf {G} \in {\mathbb {R}}^{m_{\scriptscriptstyle {L}} \times n}, \mathbf {N_{0}}\in {\mathbb {R}}^{n \times (m-m_{\scriptscriptstyle {L}})},\mathbf {v_{0}}\in {\mathbb {R}}^{m-m_{\scriptscriptstyle {L}}}.$ Hence, in the deterministic formulation, the vector $\mathbf {c}( t )$ satisfies the following ODE

16 $$\begin{aligned} \frac{\mathrm {d}}{\mathrm {d} t }\mathbf {c}( t ) = \mathbf {A} \mathbf {c}( t ) + \mathbf {b}, \end{aligned}$$

where $\mathbf {A} \in {\mathbb {R}}^{n \times n}$ and $\mathbf b \in {\mathbb {R}}^n$ are defined as follows

17a $$\begin{aligned} \mathbf {A}&:= \mathbf {\mathbf {N_{\scriptscriptstyle {L}}}G},\end{aligned}$$

17b $$\begin{aligned} \mathbf b&:= \mathbf {N_{0}}\mathbf {v_{0}}. \end{aligned}$$

Additionally we will consider the 1-norm of the vector $\mathbf {x}$, defined as $\left| \mathbf x \right| := \sum _{k=1}^{n} \left| x_k \right|$, along with the Multinomial and Poisson distributions whose probability density functions are given, respectively, by

18a $$\begin{aligned}&\mathbb N^n \times \mathbb N \times [0,1]^n \rightarrow [0,1] :\mathcal {M} \left( \mathbf {s},\xi ,\mathbf q \right) \nonumber \\&\quad := {\left\{ \begin{array}{ll} \xi ! \frac{\left( 1-\left| \mathbf q \right| \right) ^{\xi -\left| \mathbf {s} \right| }}{\left( \xi -\left| \mathbf {s} \right| \right) !} \prod _{k=1}^{n} \left( s_k !\right) ^{-1}\left( q_k\right) ^{s_k}, &{} \left| \mathbf {s} \right| \le \xi \\ 0, &{} \text {otherwise,} \end{array}\right. }\end{aligned}$$

18b $$\begin{aligned}&\mathbb N^n \times {\mathbb {R}}_{\ge 0}^n \rightarrow [0,1] :\mathcal {P} \left( \mathbf {s},\varvec{\nu }\right) := \exp {\left( -\left| \varvec{\nu } \right| \right) }\prod ^{n}_{k=1}\left( s_k !\right) ^{-1} \left( \nu _{k}\right) ^{s_k} . \end{aligned}$$

Finally, the convolution of functions $p_{1,2}(\mathbf {x}) : \mathbb {N}^n \rightarrow {\mathbb {R}}$ is defined as

19 $$\begin{aligned} p_1(\mathbf {s}) * p_2(\mathbf {s})&:= \sum _{\mathbf z} p_1(\mathbf z)p_2(\mathbf {s}- \mathbf z). \end{aligned}$$

With these definitions at hand, the forthcoming theorem presents an analytical formula for the solution of the CME in (6) with the initial probability distribution

20 $$\begin{aligned} p\left( \mathbf {s}_0,0\right) = \delta _{\varvec{\xi }} (\mathbf {s}_0) := {\left\{ \begin{array}{ll} 1,&{} \mathbf {s}_0 = \varvec{\xi }\\ 0, &{} \text {otherwise.} \end{array}\right. } \end{aligned}$$

#### **Theorem 1**

(Jahnke and Huisinga 2007) Consider the monomolecular reaction network in (14) with the initial distribution given in (20), for some $$\varvec{\xi }\in \mathbb N^n$$. Then the probability distribution at time $$ t > 0$$ is

$$\begin{aligned} p\left( \mathbf {s}^i, t \right) = \mathcal {P} \left( \mathbf {s}^i,\varvec{\nu }( t )\right) * \mathcal {M} \left( \mathbf {s}^i,\xi _1,\mathbf {q}^1( t ) \right) * \ldots * \mathcal {M} \left( \mathbf {s}^i,\xi _n,\mathbf {q}^n( t ) \right) , \end{aligned}$$

where the parameter vectors $\mathbf q^i( t )$ and $\varvec{\nu }( t )$ are the solution of the ODEs

21a $$\begin{aligned} \frac{\mathrm {d}}{\mathrm {d} t }\mathbf q^j ( t )&=\mathbf {A} \mathbf {q}^j( t ), ~ \mathbf q^j(0) = \varvec{\varepsilon }^j,\quad \forall \, j \in [1,n], \end{aligned}$$

21b $$\begin{aligned} \frac{\mathrm {d}}{\mathrm {d} t }\varvec{\nu }( t )&=\mathbf {A} \varvec{\nu }( t ) + \mathbf {b}, \quad \varvec{\nu }(0) = \varvec{0}. \end{aligned}$$

$\mathbf {A}$ and $\mathbf b$ are defined in (17) and $\varvec{\varepsilon }^j$ is the $j$th column of a $n \times n$ identity matrix.

Another useful result, Proposition 3 in (Jahnke and Huisinga 2007), is an expression for the marginal probability distribution for some species of interest.

#### **Proposition 2**

(Jahnke and Huisinga 2007) Let $p\left( \mathbf {s}, t \right)$  be the solution of the CME (6) and ${\mathcal {I}}$ be the set of subindexes of the variables $s_i$ of interest. Consider a vector $\mathbf y$ whose entries are $s_i\, \forall \ i \in {\mathcal {I}}$ and a vector $\mathbf z$ which comprises the remaining entries of $\mathbf {s}$. Then the marginal distribution,

$$\begin{aligned} F(\mathbf y, t ) := \sum _{\forall \mathbf {z}} p\left( \left( \mathbf {y},\mathbf {z} \right) , t \right) = \sum _{z_1}\sum _{z_2} \ldots p\left( \left( \mathbf {y},\mathbf {z} \right) , t \right) , \end{aligned}$$

of $p\left( \mathbf {s}, t \right)$  is given by

$$\begin{aligned} F(\mathbf y, t ) = \mathcal {P} \left( \mathbf y,\varvec{\tilde{\nu }} ( t )\right) * \mathcal {M} \left( \mathbf y,\xi _1,\tilde{\mathbf{q}}^1( t ) \right) * \ldots * \mathcal {M} \left( \mathbf y,\xi _n,\tilde{\mathbf{q}}^n( t ) \right) . \end{aligned}$$

In the equation above, the parameter vector ${\tilde{\mathbf{q}}}$ has entries $q_i\, \forall \ i \in {\mathcal {I}}$, where $\mathbf q$ is given by the solution of (21a). The definition of $\varvec{\tilde{\nu }}$ follows similarly.

In the forthcoming section, we make use of Theorem 1 and Proposition 2 (Jahnke and Huisinga 2007) to obtain an exact solution for the marginal probability distribution of the first and last state in a monomolecular reaction network. Thus, we will reduce the full system into an exact description of the species of interest.

### Marginal Probability Distribution for Selected Species

In the previous section, we showed that the ODE (16) provides a description of the numbers of molecules for each species in the reaction network (14). In this section we will obtain the analytical solution of the ODE describing the dynamics of selected species in the reaction network. To obtain the solution to such an ODE, first consider that $\mathbf {A}$ in (16) has no zero eigenvalues. Thus, we can define the error coordinate

22 $$\begin{aligned} \mathbf {e}( t ) := \mathbf {c}( t ) - \bar{\mathbf {c}}, \end{aligned}$$

where $\bar{\mathbf {c}} := -\mathbf {A}^{-1}\mathbf {b}$ represents the steady state number of molecules. In these coordinates the model (16) becomes

10a $$\begin{aligned} \frac{\mathrm {d}}{\mathrm {d} t }\mathbf {e}( t )&=\mathbf {A} \mathbf {e}( t ),\quad \mathbf {e}(0) = \mathbf {e}_0, \end{aligned}$$

10b $$\begin{aligned} \mathbf y( t )&=\mathbf {C}\mathbf {e}( t ),\qquad \qquad \qquad \end{aligned}$$

where $\mathbf C$ selects the entries of interest in $\mathbf {e}( t )$. As described in Proposition 2, the marginal probability distribution for selected species is given by the convolution of probability distributions. These probability distributions depend on the parameters ${\tilde{\mathbf{q}}}^j( t )$ and $\varvec{\tilde{\nu }} ( t )$, which are solutions of the differential equations in (21). There, $\mathbf {A}$ is as in (10a) above. In Proposition 1 and Corollary 1, we derived expressions for such solutions. With the appropriate variables, the solutions for ${\tilde{\mathbf{q}}}^j( t )$ and $\varvec{\tilde{\nu }} ( t )$ are given by

23a $$\begin{aligned} {\tilde{\mathbf{q}}}^j( t )&=\sum _{\ell ={1}}^n\vartheta _{\ell }\exp (\lambda _\ell t)\mathbf {m}^j (\lambda _\ell ),\end{aligned}$$

23b $$\begin{aligned} \varvec{\tilde{\nu }} ( t )&=-\sum _{\ell ={1}}^n\vartheta _{\ell }\exp (\lambda _\ell t)\mathbf {M} (\lambda _\ell )\varvec{\bar{\nu }} + \mathbf {C} \varvec{\bar{\nu }}. \end{aligned}$$

where $\vartheta _{\ell }$ and $\mathbf {M} (\lambda _\ell )$ are as in (12); the vector $\mathbf {m}^j(\lambda _\ell )$ is the $j$th column of $\mathbf {M} (\lambda _\ell )$; and $\varvec{\bar{\nu }}$ is the steady state of (21b), i.e., $\varvec{\bar{\nu }} = -\mathbf {A}^{-1}\mathbf {b}$. In case $\mathbf {A}$ has repeated eigenvalues, we refer the interested reader to the supplemental online material (SOM) for the derivation of analogue expressions to (23). In the following section, we apply these results to an unbranched chain of monomolecular reactions.

### Unbranched Monomolecular Reaction Chain with Synthesis and Degradation

In this section we focus on a more specific class of reaction networks described by the following reactions

24a $$\begin{aligned}&S_1 \mathop {\rightleftharpoons }\limits _{k_{\mathrm {b} 1}}^{k_{\mathrm {f} 1}} S_2 \mathop {\rightleftharpoons }\limits _{k_{\mathrm {b} 2}}^{k_{\mathrm {f} 2}} \ldots \mathop {\rightleftharpoons }\limits _{k_{\mathrm {b} n-1}}^{k_{\mathrm {f} n-1}} S_n,\end{aligned}$$

24b $$\begin{aligned}&S_i \xrightarrow {k_{\mathrm {d} i}} 0,\end{aligned}$$

24c $$\begin{aligned}&0 \xrightarrow {k_{\mathrm {s} i}} S_{i}. \end{aligned}$$

For this reaction network, the matrices $\mathbf {A}$ and $\mathbf b$ in (16) are

25a $$\begin{aligned} \mathbf {A}&=\begin{pmatrix} -\left( k_{\mathrm {f} 1} + k_{\mathrm {d} 1} \right) &{}\quad \!\! k_{\mathrm {b} 1} &{}\quad \!\! 0 &{}\quad \!\! \ldots &{}\quad \!\! 0 &{}\quad \!\! 0&{}\quad \!\! 0\\ k_{\mathrm {f} 1} &{}\quad \!\! -\left( k_{\mathrm {b} 1} + k_{\mathrm {f} 2} + k_{\mathrm {d} 2}\right) &{}\quad \!\! k_{\mathrm {b} 2}&{}\quad \!\! \vdots &{}\quad \!\! 0&{}\quad \!\! 0&{}\quad \!\! 0\\ \vdots &{}\quad \!\! \vdots &{}\quad \!\! \vdots &{}\quad \!\! \ddots &{}\quad \!\! \ddots &{}\quad \!\! \ddots &{}\quad \!\! \vdots \\ 0 &{}\quad \!\! 0 &{}\quad \!\! 0&{}\quad \!\! \ldots &{}\quad \!\! k_{\mathrm {f} n-2}&{}\quad \!\! -\left( k_{\mathrm {b} n-2} + k_{\mathrm {f} n-1} + k_{\mathrm {d} n-1}\right) &{}\quad \!\! k_{\mathrm {b} n-1}\\ 0 &{}\quad \!\! 0 &{}\quad \!\! 0&{}\quad \!\! \ldots &{}\quad \!\! 0&{}\quad \!\! k_{\mathrm {f} n-1}&{}\quad \!\! -\left( k_{\mathrm {b} n-1} + k_{\mathrm {d} n} \right) \\ \end{pmatrix},\end{aligned}$$

25b $$\begin{aligned} \mathbf b&=\begin{pmatrix} k_{\mathrm {s} 1}&\quad \!\! k_{\mathrm {s} 2}&\quad \!\! \ldots&\quad \!\! k_{\mathrm {s} n} \end{pmatrix}^T. \end{aligned}$$

As noted in Barrio et al. (2013), the matrix $\mathbf {A}$ above is a real tridiagonal matrix with positive off diagonal elements and, hence, it is similar to an Hermitian matrix (Veselić 1979; Bernstein 2009, p. 359). This implies that $\mathbf {A}$ has real, simple eigenvalues. In addition, the Gerschgorin disk of the $i$th column lies in the left hand complex plane at a distance $k_{\mathrm {d} i}$ of the imaginary axis. However, for large dimensions of $\mathbf {A}$, the numerical computation of the eigenvalues of $\mathbf {A}$ might not yield real eigenvalues, due to accumulation of numerical errors during the computations (Wilkinson 1984).

We note as well, that for most kinetic parameter values, the columns of $\mathbf {A}$ are linearly independent. Hence $\mathbf {A}$ has no zero eigenvalue and we can make use of Proposition 1 and Corollary 1 to obtain closed form solutions for the first and last states of the reaction network.

The matrix $\mathbf {M}(\zeta )$ in (12a) is composed of some columns of the adjugate matrix of $\zeta \mathbf {I-A}$. In general, we would need to compute as many cofactors of $\zeta \mathbf {I-A}$ as its number of elements to construct this adjugate matrix. However, for a tridiagonal matrix there is a recursive algorithm that obtains elements of its inverse, from which the adjugate matrix of $\zeta \mathbf {I-A}$ can be easily computed and at a lower computational cost.

#### **Proposition 3**

(Usmani 1994) Let $\mathbf {T}$ be a square, $n\times n$, tridiagonal, invertible matrix; that is $T_{ij}=0$ for $\left| i-j \right| >1$. Then the elements of the inverse of $\mathbf T$ are given by

26 $$\begin{aligned} \left( T^{-1}\right) _{ij} = (\theta _n)^{-1}\times {\left\{ \begin{array}{ll} (-1)^{i+j} \theta _{i-1} \phi _{j+1} \prod _{k=i}^{j-1} T_{k,k+1}, &{} i<j, \\ \theta _{i-1}\phi _{i+1}, &{} i=j,\\ \left( -1 \right) ^{i+j} \theta _{j-1}\phi _{i+1} \prod _{k=j+1}^{i} T_{k,k-1}, &{} i>j, \end{array}\right. } \end{aligned}$$

where $\theta _{i}$ and $\phi _{i}$ satisfy the recursions

$$\begin{aligned} \theta _i&=T_{ii} \theta _{i-1} - T_{i,i+1}T_{i-1,i}\theta _{i-2}, \quad \theta _{-1} = 0, \quad \theta _{0} = 1, \quad i \in [1,n],\\ \phi _{i}&=T_{ii}\phi _{i+1}-T_{i,i+1}T_{i+1,i}\phi _{i+2}, \quad \phi _{n+2} = 0, \quad \phi _{n+1} = 1, \quad i \in [1,n]. \end{aligned}$$

Of note, the $\theta _{i}$s above are the principal minors of $\mathbf T$, thus $\theta _n = \det \left( \mathbf T\right)$. Hence the adjugate matrix of $\mathbf T$ can be obtained by multiplying the expression in (26) by $\theta _n$.

In the following section, we revisit the unbranched monomolecular reaction network studied in this section, while considering all the synthesis and degradation rates to be equal to zero. In that case, $\mathbf {A}$ is singular. Hence the equilibrium point of such a network has to be derived in an alternative way.

### Unbranched Monomolecular Reaction Chain

In this section we focus on the following reaction chain

24a $$\begin{aligned}&S_1 \mathop {\rightleftharpoons }\limits _{k_{\mathrm {b} 1}}^{k_{\mathrm {f} 1}} S_2 \mathop {\rightleftharpoons }\limits _{k_{\mathrm {b} 2}}^{k_{\mathrm {f} 2}} \ldots \mathop {\rightleftharpoons }\limits _{k_{\mathrm {b} n-1}}^{k_{\mathrm {f} n-1}} S_n. \end{aligned}$$

For this case, the stoichiometric matrix and reaction rate vector in (15) have the form

27a $$\begin{aligned} \mathbf {N_{\scriptscriptstyle {L}}}&=\begin{pmatrix} -1&{}\quad 0 &{}\quad \ldots &{}\quad 0\\ 1 &{}\quad -1&{}\quad \ddots &{}\quad \vdots \\ 0 &{}\quad 1&{}\quad \ddots &{}\quad \vdots \\ \vdots &{}\quad \ddots &{}\quad \ddots &{}\quad \vdots \\ 0&{}\quad 0&{}\quad \ddots &{}\quad -1\\ 0&{}\quad 0&{}\quad \ldots &{}\quad 1 \end{pmatrix} \in \mathbb {N}^{n\times (n-1)},\end{aligned}$$

27b $$\begin{aligned} \mathbf G&=\begin{pmatrix} k_{\mathrm {f} 1} &{}\quad -k_{\mathrm {b} 1} &{}\quad 0 &{}\quad \ldots &{}\quad 0 &{}\quad 0 \\ 0 &{}\quad k_{\mathrm {f} 2}&{}\quad -k_{\mathrm {b} 2}&{}\quad \ddots &{}\quad 0&{}\quad 0\\ \vdots &{}\quad \ddots &{}\quad \ddots &{}\quad \ddots &{}\quad \ddots &{}\quad \vdots \\ 0&{}\quad 0&{}\quad 0&{}\quad \ldots &{}\quad k_{\mathrm {f} n-1}&{}\quad -k_{\mathrm {b} n-1} \end{pmatrix}\in {\mathbb {R}}^{(n-1)\times n}. \end{aligned}$$

Whence the matrix $\mathbf {A}$ in (16) becomes

28 $$\begin{aligned} \mathbf {A} = \begin{pmatrix} -k_{\mathrm {f} 1} &{}\quad k_{\mathrm {b} 1} &{}\quad 0 &{}\quad \ldots &{}\quad 0 &{}\quad 0&{}\quad 0\\ k_{\mathrm {f} 1} &{}\quad -\left( k_{\mathrm {b} 1} + k_{\mathrm {f} 2} \right) &{}\quad k_{\mathrm {b} 2}&{}\quad \vdots &{}\quad 0&{}\quad 0&{}\quad 0\\ \vdots &{}\quad \ddots &{}\quad \ddots &{}\quad \ddots &{}\quad \ddots &{}\quad \ddots &{}\quad \vdots \\ 0 &{}\quad 0 &{}\quad 0&{}\quad \ldots &{}\quad k_{\mathrm {f} n-2}&{}\quad -\left( k_{\mathrm {b} n-2} + k_{\mathrm {f} n-1} \right) &{}\quad k_{\mathrm {b} n-1}\\ 0 &{}\quad 0 &{}\quad 0&{}\quad \ldots &{}\quad 0&{}\quad k_{\mathrm {f} n-1}&{}\quad -k_{\mathrm {b} n-1}\\ \end{pmatrix},\nonumber \\ \end{aligned}$$

and the vector $\mathbf b$ in (16) is the zero vector. Since both $\mathbf {N_{\scriptscriptstyle {L}}}$ and $\mathbf G$ in (27) are rank deficient, the matrix $\mathbf {A}$ is also rank deficient. The forthcoming proposition provides a formula for the steady state of (24a), while the details of its derivation can be found in Appendix.

#### **Proposition 4**

Consider the system (16) along with the definitions in (27). This ODE has a unique fixed point ${\bar{\mathbf {\mathbf {c}}}}$, whose $k$th entry is

29 $$\begin{aligned} \bar{c}_k&=\frac{\left( \prod _{i=k}^{n-1} k_{\mathrm {b} i} \right) \left( \prod _{i=1}^{k-1} k_{\mathrm {f} i} \right) }{\sum _{j=1}^{n} \left( \prod _{i=j}^{n-1} k_{\mathrm {b} i} \right) \left( \prod _{i=1}^{j-1} k_{\mathrm {f} i} \right) } \sum _{i=1}^{n} c_i(0). \end{aligned}$$

In the proof of the proposition above, we made use of the null spaces of the stoichiometric matrix $\mathbf {N_{\scriptscriptstyle {L}}}$. For a more detailed explanation and use of such null spaces, we refer the interested reader to Palsson (2006) and to López-Caamal et al. (2014) for an application of such methods to obtain the equilibrium points of reaction networks resembling a class of positive feedback loops.

We note that the matrix $\mathbf {A}$ in (28) is real, Metzler, tridiagonal and that the elements of its columns add up to zero. As it is a rank-deficient matrix, it has a zero eigenvalue. Moreover, the Gershgorin disks of every column lie in the closed left hand side of the complex plane. As this matrix is also similar to an Hermitian matrix (Veselić 1979), all the eigenvalues are simple and real and, consequently, the zero eigenvalue is unique. Despite $\mathbf {A}$ being singular, we can use the formula for the equilibrium point in (29) to evaluate the marginal probability distribution of Proposition 2, via the expressions in (23).

The dynamical properties of the reaction topology in (24a) with $k_{\mathrm {b} i} = 0$ was studied in Oyarzún et al. (2009), where the authors show the existence of optimal principles behind the dynamics of metabolic regulation in the deterministic framework. In the next section, we analyse a last reaction network topology in which the first and last species in the chain of reactions react to each other, therefore creating a ring of reactions.

### Ring of Monomolecular Reactions

In this section we consider the reaction network

24a $$\begin{aligned}&S_1 \mathop {\rightleftharpoons }\limits _{k_{\mathrm {b} 1}}^{k_{\mathrm {f} 1}} S_2 \mathop {\rightleftharpoons }\limits _{k_{\mathrm {b} 2}}^{k_{\mathrm {f} 2}} \ldots \mathop {\rightleftharpoons }\limits _{k_{\mathrm {b} n-1}}^{k_{\mathrm {f} n-1}} S_n, \end{aligned}$$

30 $$\begin{aligned}&S_n \mathop {\rightleftharpoons }\limits _{k_{\mathrm {b} n}}^{k_{\mathrm {f} n}} S_1.\qquad \end{aligned}$$

For this reaction network, the stoichiometric matrix and reaction rate vector in (15) have the form

31a $$\begin{aligned} \mathbf {N_{\scriptscriptstyle {L}}}&=\begin{pmatrix} -1&{}\quad 0 &{}\quad \ldots &{}\quad 0&{}\quad 1\\ 1 &{}\quad -1&{}\quad \ddots &{}\quad \vdots &{}\quad 0\\ 0 &{}\quad 1&{}\quad \ddots &{}\quad \vdots &{}\quad 0\\ \vdots &{}\quad \ddots &{}\quad \ddots &{}\quad \vdots &{}\quad 0\\ 0&{}\quad 0&{}\quad \ddots &{}\quad -1&{}\quad 0\\ 0&{}\quad 0&{}\quad \ldots &{}\quad 1&{}\quad -1 \end{pmatrix} \in \mathbb {N}^{n\times n},\end{aligned}$$

31b $$\begin{aligned} \mathbf G&=\begin{pmatrix} k_{\mathrm {f} 1} &{}\quad -k_{\mathrm {b} 1} &{}\quad 0 &{}\quad \ldots &{}\quad 0 &{}\quad 0 \\ 0 &{}\quad k_{\mathrm {f} 2}&{}\quad -k_{\mathrm {b} 2}&{}\quad \ddots &{}\quad 0&{}\quad 0\\ \vdots &{}\quad \ddots &{}\quad \ddots &{}\quad \ddots &{}\quad \ddots &{}\quad \vdots \\ 0&{}\quad 0&{}\quad 0&{}\quad \ldots &{}\quad k_{\mathrm {f} n-1}&{}\quad -k_{\mathrm {b} n-1}\\ -k_{\mathrm {b} n} &{}\quad 0&{}\quad 0&{}\quad \ldots &{}\quad 0&{}\quad k_{\mathrm {f} n} \end{pmatrix}\in {\mathbb {R}}^{n\times n}. \end{aligned}$$

We will further assume that $\mathbf G$ is invertible. With the definitions above, $\mathbf {A}$ in (16) has the form

32 $$\begin{aligned} \mathbf {A} = \begin{pmatrix} - \left( k_{\mathrm {f} 1} + k_{\mathrm {b} n}\right) &{}\quad \! k_{\mathrm {b} 1} &{}\quad \! 0 &{}\quad \! \ldots &{}\quad \! 0 &{}\quad \! 0&{}\quad \! k_{\mathrm {f} n}\\ k_{\mathrm {f} 1} &{}\quad \! -\left( k_{\mathrm {b} 1} + k_{\mathrm {f} 2} \right) &{}\quad \! k_{\mathrm {b} 2}&{}\quad \! \vdots &{}\quad \! 0&{}\quad \! 0&{}\quad \! 0\\ \vdots &{}\quad \! \vdots &{}\quad \! \vdots &{}\quad \! \ddots &{}\quad \! \ddots &{}\quad \! \ddots &{}\quad \! \vdots \\ 0 &{}\quad \! 0 &{}\quad \! 0&{}\quad \! \ldots &{}\quad \! k_{\mathrm {f} n-2}&{}\quad \! -\left( k_{\mathrm {b} n-2} + k_{\mathrm {f} n-1} \right) &{}\quad \! k_{\mathrm {b} n-1}\\ k_{\mathrm {b} n} &{}\quad \! 0 &{}\quad \! 0&{}\quad \! \ldots &{}\quad \! 0&{}\quad \! k_{\mathrm {f} n-1}&{}\quad \! -\left( k_{\mathrm {b} n-1} + k_{\mathrm {f} n}\right) \\ \end{pmatrix},\!\!\!\!\nonumber \\ \end{aligned}$$

By following the lines of Proposition 4, we can show that the equilibrium point of this system is given by

33 $$\begin{aligned} \bar{c}_k = \frac{\sum _{\ell =1}^n w_{k\ell }}{\sum _{j=1}^{n}\sum _{\ell =1}^{n} w_{j\ell }}\sum _{i=1}^{n} c_i(0), \end{aligned}$$

Here $w_{j\ell }$ is the $j\ell$th element of $\mathbf G^{-1}$. The key point of such a derivation is to note that both the left and right null space of $\mathbf {N_{\scriptscriptstyle {L}}}$ are nontrivial and spanned by $\varvec{1} ^T$ and $\varvec{1}$, respectively.

As $\mathbf {N_{\scriptscriptstyle {L}}}$ in (31) is rank deficient, $\mathbf {A}$ has a zero eigenvalue. As in the previous section, we can conclude that all eigenvalues have nonpositive real part by analysing the Gershgorin disks of each column. However, it is not easy to conclude whether or not the eigenvalues of such a matrix are simple or real, as they depend on particular kinetic parameter values. Depending on the nature of the eigenvalues of $\mathbf {A}$, we have to choose between the expressions in (23) or those in the SOM to evaluate the marginal probability distribution described in Proposition 2. In the following section we apply our results to particular reaction networks.

## Case Studies

In this section, first, we analyse a protein autoactivation mechamism modelled by nonlinear propensities. Later, we obtain the marginal probability density function for the first and last species in a monomolecular reaction network. In such network, we compare the computational load required to derive such a probability density function by means of (i) using the stochastic simulation algorithms to generate independent trajectories, complemented with further statistical analysis and (ii) the evaluation of the formula provided in Proposition 2 by means of the expressions for ${\tilde{\mathbf{q}}}^j$ and $\varvec{\tilde{\nu }}$ in (23). Finally, we analyse a four-species ring that describes the opening of calcium channels in the sarcoplasmic reticulum mediated by ryanodine receptors.

### Protein Autoactivation Mechanism

Consider the following protein autoactivation mechanism

34 $$\begin{aligned} P + A \mathop {\rightleftharpoons }\limits _{k_{\mathrm b1}}^{k_{\mathrm f1}} 2A, \quad P \xrightarrow {k_{\mathrm d1}} 0, \quad A \xrightarrow {k_{\mathrm d2}} 0, \end{aligned}$$

where $P$ represents a protein and $A$ its active form. We will focus on the derivation of the probability of having a certain number of active proteins $A$, given an initial condition.

By selecting the vector order $\mathbf {s}= ( \text {Number of molecules of } P , \text {Number of} \text {molecules of A} )^T$, the stoichiometric matrix is

$$\begin{aligned} \mathbf N = \begin{pmatrix} -1 &{}\quad 1&{}\quad -1&{}\quad 0\\ 1&{}\quad -1&{}\quad 0&{}\quad -1 \end{pmatrix} \end{aligned}$$

and the propensity vector becomes (cfr. Table 1)

$$\begin{aligned} \mathbf {a}(\mathbf {s}( t )) = \begin{pmatrix} k_{\mathrm f1} s_1( t )s_2( t )&\frac{k_{\mathrm b1}}{2}s_2( t )(s_2( t ) -1)&k_{\mathrm d1} s_1( t )&k_{\mathrm d2} s_2( t ) \end{pmatrix} ^T. \end{aligned}$$

We note that the reaction network in (34) is a closed system, hence the possible combinations of number of molecules is finite and determined by the initial condition. For simplicity, let us assume that we initially have 2 molecules of $P$ and 1 molecule of $A$. Let us consider that the set $\mathbf {S}$ in (4) is ordered as follows

35 $$\begin{aligned} \mathbf {S}= \left\{ \begin{pmatrix} 2 \\ 1 \end{pmatrix} , \begin{pmatrix} 1 \\ 2\end{pmatrix} , \begin{pmatrix} 3 \\ 0\end{pmatrix} , \begin{pmatrix} 1 \\ 1\end{pmatrix} , \begin{pmatrix} 2 \\ 0\end{pmatrix} , \begin{pmatrix} 0 \\ 3\end{pmatrix} , \begin{pmatrix} 0 \\ 2\end{pmatrix} , \begin{pmatrix} 0 \\ 1\end{pmatrix} , \begin{pmatrix} 1 \\ 0\end{pmatrix} , \begin{pmatrix} 0 \\ 0 \end{pmatrix} \right\} . \end{aligned}$$

With this order, along with the parameters values $\{k_{\mathrm f1},k_{\mathrm b1},k_{\mathrm s1},k_{\mathrm s2}\} = \{0.15,0.1,0.13,0.2\} [\mathrm s^{-1}]$, the matrix ${\mathcal {A}}$ in (8) is

36 $$\begin{aligned} {\mathcal {A}} = \frac{1}{100}\begin{pmatrix} -76&{}\quad 10&{}\quad 0&{}\quad 0&{}\quad 0&{}\quad 0&{}\quad 0&{}\quad 0&{}\quad 0&{}\quad 0\\ 30&{}\quad -93&{}\quad 0&{}\quad 0&{}\quad 0&{}\quad 30&{}\quad 0&{}\quad 0&{}\quad 0&{}\quad 0\\ 0&{}\quad 0&{}\quad -39&{}\quad 0&{}\quad 0&{}\quad 0&{}\quad 0&{}\quad 0&{}\quad 0&{}\quad 0\\ 26&{}\quad 40&{}\quad 0&{}\quad -48&{}\quad 0&{}\quad 0&{}\quad 10&{}\quad 0&{}\quad 0&{}\quad 0\\ 20&{}\quad 0&{}\quad 39&{}\quad 0&{}\quad -26&{}\quad 0&{}\quad 0&{}\quad 0&{}\quad 0&{}\quad 0\\ 0&{}\quad 30&{}\quad 0&{}\quad 0&{}\quad 0&{}\quad -90&{}\quad 0&{}\quad 0&{}\quad 0&{}\quad 0\\ 0&{}\quad 13&{}\quad 0&{}\quad 15&{}\quad 0&{}\quad 60&{}\quad -50&{}\quad 0&{}\quad 0&{}\quad 0\\ 0&{}\quad 0&{}\quad 0&{}\quad 13&{}\quad 0&{}\quad 0&{}\quad 40&{}\quad -20&{}\quad 0&{}\quad 0\\ 0&{}\quad 0&{}\quad 0&{}\quad 20&{}\quad 26&{}\quad 0&{}\quad 0&{}\quad 0&{}\quad -13&{}\quad 0\\ 0&{}\quad 0&{}\quad 0&{}\quad 0&{}\quad 0&{}\quad 0&{}\quad 0&{}\quad 20&{}\quad 13&{}\quad 0\\ \end{pmatrix},\!\!\!\nonumber \\ \end{aligned}$$

which has simple and real eigenvalues. As we are interested in the probability of having a certain number of molecules of $A$, let

$$\begin{aligned} \mathbf {y}( t ) := \left( \mathrm {Probability}(s_2 = 0)\quad \mathrm {Probability}(s_2 \!=\! 1)\quad \mathrm {Probability}(s_2 \!=\! 2)\,\,\,\,\, \mathrm {Probability} (s_2 = 3)\right) ^T, \end{aligned}$$

which can be computed as $\mathbf y( t ) \!= \!\mathbf {C} \mathbf p ( t )$, were $\mathbf p( t )$ is the solution of the CME in (10). To construct $\mathbf C$ we note that to obtain $\mathrm {Probability}(s_2 = 0)$, for instance, we have to add the probabilities of being in states $\mathbf {s}^{3}, \mathbf {s}^{5}, \mathbf {s}^{9}$, and $\mathbf {s}^{10}$, following the order of the states given in (35). Hence in the first row of $\mathbf C$, we fill in a 1 for the third, fifth, ninth, and tenth position and leave a zero in the remaining elements, so as the multiplication of this row by $\mathbf p( t )$ yields the desired sum. By continuing this procedure for all rows, the matrix $\mathbf C$ becomes

$$\begin{aligned} \mathbf C = \begin{pmatrix} 0&{}\quad 0&{}\quad 1&{}\quad 0&{}\quad 1&{}\quad 0&{}\quad 0&{}\quad 0&{}\quad 1&{}\quad 1\\ 1&{}\quad 0&{}\quad 0&{}\quad 1&{}\quad 0&{}\quad 0&{}\quad 0&{}\quad 1&{}\quad 0&{}\quad 0\\ 0&{}\quad 1&{}\quad 0&{}\quad 0&{}\quad 0&{}\quad 0&{}\quad 1&{}\quad 0&{}\quad 0&{}\quad 0\\ 0&{}\quad 0&{}\quad 0&{}\quad 0&{}\quad 0&{}\quad 1&{}\quad 0&{}\quad 0&{}\quad 0&{}\quad 0 \end{pmatrix}. \end{aligned}$$

**Fig. 1:**
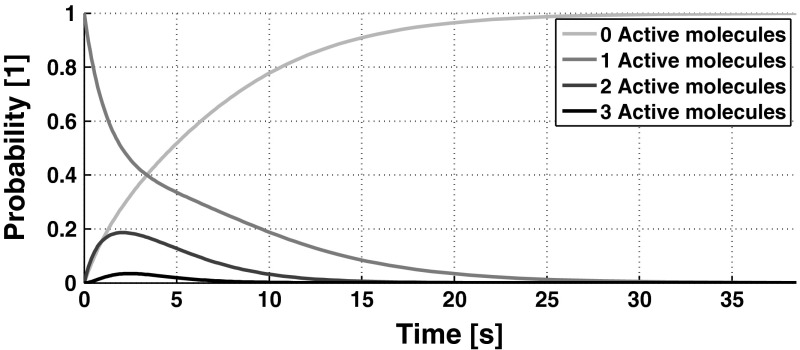
Probabilities of having a specific number of active molecules, for the reaction network in (34), with the parameters described in Example 4.1.

Assuming that with probability 1 the initial state is $\mathbf {s}^1$, we can avail of Corollary 1 to obtain the solution for $\mathbf {y}( t )$. Due to space constrains, we omit the explicit formula for $\mathbf y( t )$ and depict in Fig. 1 such a solution. It is worth noting that, for the simple reaction network with only 3 initial molecules studied in the foregoing example, we have associated a CME with 10 states. In turn, if we wanted to analyse the very same system but now with 10 initial molecules for each species, the CME would have 231 states. By increasing the number of initial molecules, the number of states of the CME will quickly increase, hence rendering infeasible its analytical approach. Especially when the number of reactions is large. In the following section, we study an unbranched monomolecular reaction chain.

### Computational Load for an Unbranched Monomolecular Reaction Chain

Now, we focus on an unbranched monomolecular reaction network with synthesis and degradations for all species, as shown in (24a,b,c) of Sect. 3.3. That is, we consider the following reaction network

24a $$\begin{aligned}&S_1 \mathop {\rightleftharpoons }\limits _{k_{\mathrm {b} 1}}^{k_{\mathrm {f} 1}} S_2 \mathop {\rightleftharpoons }\limits _{k_{\mathrm {b} 2}}^{k_{\mathrm {f} 2}} \ldots \mathop {\rightleftharpoons }\limits _{k_{\mathrm {b} n-1}}^{k_{\mathrm {f} n-1}} S_n, \end{aligned}$$

24b $$\begin{aligned}&S_i \xrightarrow {k_{\mathrm {d} i}} 0,\quad \end{aligned}$$

24c $$\begin{aligned}&0 \xrightarrow {k_{\mathrm {s} i}} S_{i}.\quad \end{aligned}$$

There we concluded that the eigenvalues of the matrix $\mathbf {A}$ in (16) are simple and real. Hence, we may use the closed form solutions for ${\tilde{\mathbf{q}}}^j$ and $\mathbf {\varvec{\tilde{\nu }}}$ given in (23) and Proposition 2, so as to obtain the marginal probability distribution for the first, $S_1$, and last species, $S_n$. Additionally, as $\mathbf {A}$ in (25a) is a tridiagonal matrix, we avail of Proposition 3 to compute the cofactors that compose $\mathbf {M}(\lambda _\ell )$ in (12a).

We implemented these expressions in a 3.2 GHz Quad-Core Intel Xeon computer with 16GB of RAM. Our script was coded in MATLAB  R2012b. To benchmark the performance of our approach, we also implemented the SSA and varied the number of species $n$ in the reaction network (24). We chose the parameters $k_{\mathrm {f} i}, k_{\mathrm {b} i}, k_{\mathrm {d} i}, k_{\mathrm {s} i}$ to be randomly drawn from the uniform distribution $U[0,1]$. In turn, the initial numbers of molecules in $\mathbf {c}(0)$ were also uniformly randomly drawn from the uniform distribution $U[1,10]$.

Once determined the reaction chain length, parameters and initial conditions, we ran the SSA algorithm $10^3$ and $10^5$ times, respectively. By using the eigenvalues of $\mathbf {A}$, we estimated the time for the probability distributions to reach equilibrium and performed simulations until such a time was reached. That way we capture all transient probability distributions. The final time is $t_\mathrm{f} = 6/|\lambda _{\mathrm {max}}|$, where $\lambda _{\mathrm {max}}$ is the largest, non-zero eigenvalue of $\mathbf {A}$. Then, we extracted the marginal probability distribution, and registered the computational time required by this numerical approach, denoted by $t_{\mathrm {NUM}}$. In turn, we tracked the time required for the evaluation of our methodology, denoted by $t_{\mathrm A}$. To compare both computational loads, we evaluate the expression

37 $$\begin{aligned} { r = \log _{10} \left( \frac{t_{\mathrm {NUM}}-t_{\mathrm A}}{t_{\mathrm A}} \right) ,} \end{aligned}$$

and summarise this comparison in Table 3 for every chosen chain length $n$.

**Table 3:** Comparison of the computational time required by the implementation of the SSA and the formulas described in Sect. 3.3

Number of species $n$	$r$ with $10^3$ SSA runs	$r$ with $10^5$ SSA runs
5	2.4183	4.2632
10	2.4904	4.3132
15	2.5589	4.5446
20	2.4758	4.7313
25	2.7144	4.8895
30	2.6191	4.7968
35	2.6336	4.6733
40	2.7353	4.4892
45	3.0813	4.8945
50	3.5927	4.7001

The comparisson factor $r$ is defined in (37)

Please note that there is no clear trend for $r$ as $n$ increases, owing to the fact that for every comparison made with a certain number of species and number of realisations of the SSA a random set of parameters and initial conditions was drawn. We note that, regardless of the parameters used for simulation, the computational savings in this example are on the orders of magnitude of the SSA repetitions. From our computational experiments (data not shown) we noted that the evaluation of the analytical expressions to obtain the desired marginal probability distributions takes about the same time as a single run of the SSA algorithm (0.01–1 s).

Furthermore, we note that our approach is based on an exact solution of the marginal probability distributions. Hence, in addition to computational savings, the accuracy obtained with our methodology outperforms that obtained by any numerical approach.

Now, we focus on the effect that the chain-length has on the efficiency of our methodology. Toward this end, we assigned all the kinetic parameters to be $$\{k_{\mathrm {f} i},k_{\mathrm {b} i},k_{\mathrm {d} i},k_{\mathrm {s} i}\}$$ = $$\{1.396,0.465,0.851,0.398\} [\mathrm s^{-1}]\,\forall \, i \in [1,n]$$, 10 molecules for each species at $$ t = 0$$, and varied the number of species in the chain $$n$$. For each $$n$$, we performed $$10^3$$ runs of different simulation algorithms: namely, (i) the SSA, (ii) the NRM (Gibson and Bruck 2000), (iii) ODM (Cao et al. 2004), and (iv) a hybrid stochastic simulation algorithm (Liu et al. 2012); analysed their outcome statistically; and compared the computational time required with that of our methodology.

**Fig. 2:**
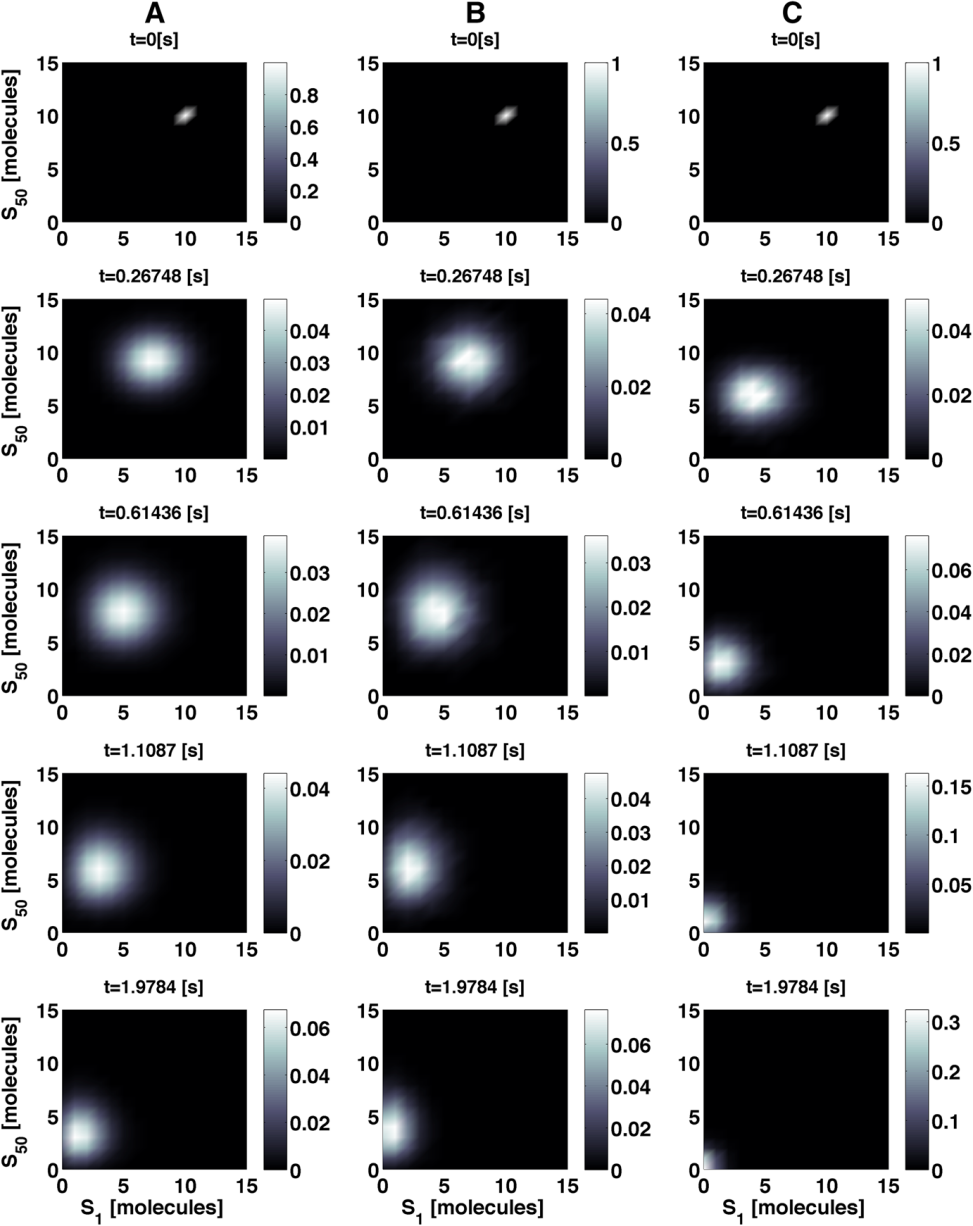
Marginal probability distribution for $S_1$ and $S_{50}$. The column **a** depicts the evaluation of the analytical solution as time progresses, whereas column **b** shows the marginal probability distribution as obtained from $10^3$ independent runs of the SSA. Likewise, column **c** shows the outcome of from $10^3$ runs of the hybrid simulation algorithm. The parameter values are $\{k_{\mathrm {f} i},k_{\mathrm {b} i},k_{\mathrm {d} i},k_{\mathrm {s} i}\} = \{1.396,0.465,0.851,0.398\}[\mathrm s^{-1}]\, \forall \, i \in [1,n]$ and 10 initial molecules for each species

**Fig. 3:**
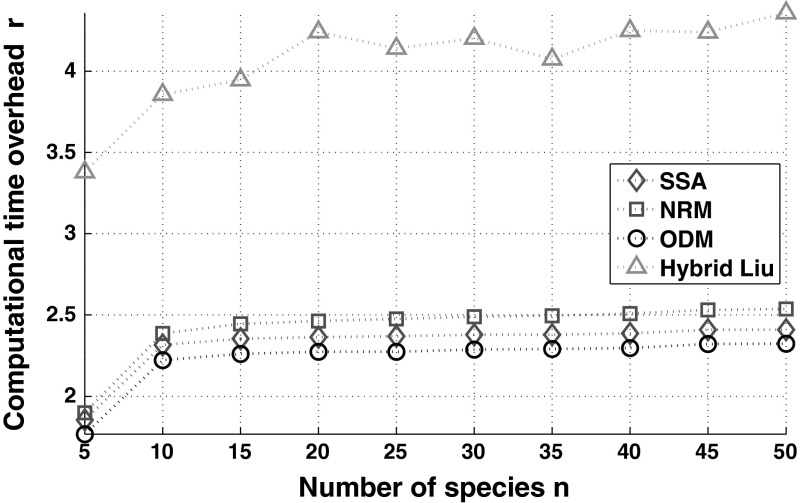
Comparison of the computational time overheads recorded from $10^3$ independent runs of the SSA, NRM, ODM, and a hybrid stochastic simulation algorithm, as compared with our methodology (i.e. formulae described in Sect 3.3). The parameters values are those used in Fig. 2. The variable $r$ is defined in (37)

For exemplification, we present in Fig. 2 the outcome of the analytical solution and compare it with the analysis of the data arising from $10^3$ runs of Stochastic Simulation Algorithm, SSA and the hybrid stochastic simulation algorithm in Liu et al. (2012). In turn, Fig. 3 summarises the benchmark of the computational time. For this example, we note that the SSA outperforms our implementation of the NRM. This observation aligns with remarks in Cao et al. (2004), which state that the cost of maintaining the computational structures required for the NRM are high.

We note that the hybrid method is around one order of magnitude slower than the the rest of the algorithms. This might be consequence of the fact that, for the reaction under analysis, there might not exist a time-scale separation required by hybrid simulation methods.

**Fig. 4:**
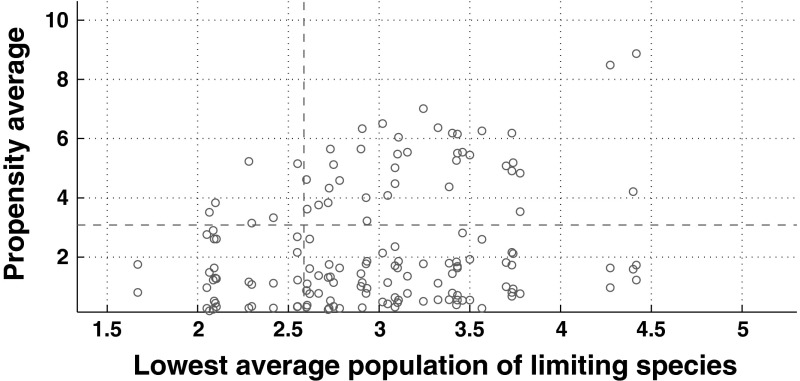
Average reaction propensity as a function of the population average of its reactants in a long SSA run of the reaction network (24). Each circle represents the average of the reaction propensity for each reaction as a function of the average of its limiting species. We note that most of the points are gathered in one region. The discontinuous lines delimit the regions for slow and fast reactions, required for the hybrid simulation method. The reactions in the region of low population average and low propensity average are considered slow reactions; whereas the rest of the reactions are considered as fast. Here, we consider the case in which we have 50 different species $(n=50)$ and the parameters values are those used in Fig. 2

To see this, we depict in Fig. 4 the number of reactions’ firings as a function of the population average of the limiting species, for each reaction. There one can observe that all the reactions are clustered into one single group and hence it is difficult to classify the reactions as fast or slow. We refer the interested reader to Liu et al. (2012, Sec. III B) for a discussion of a suitable time-scale separation for using this hybrid simulation method.

Up to now, we have compared the computational time required to obtain the solution to the CME via our analytical approach and the computational time required for obtaining approximations of such solutions via different stochastic simulation methods. Now we adopt a converse perspective and compare the accuracy of the simulation-based methods with that of our closed-form expressions for the solution of the CME.

To do so, we choose $\eta$ time-points along the transient obtained by the simulation-based methods and compare the resulting marginal probability distribution $F_\mathrm{S} (\mathbf {y}, t )$ with our exact, analytical result $F_\mathrm{A} (\mathbf y, t )$. We evaluate such a comparison with the expression

38 $$\begin{aligned} \mathrm {Error} = \log _{10} \left( \frac{1}{\eta } \sum _{i = 1}^{\eta } \sum _{\forall ~\mathbf y} \left| F_\mathrm{A} (\mathbf y, t _i) -F_\mathrm{S} (\mathbf {y}, t _i) \right| \right) . \end{aligned}$$

**Fig. 5:**
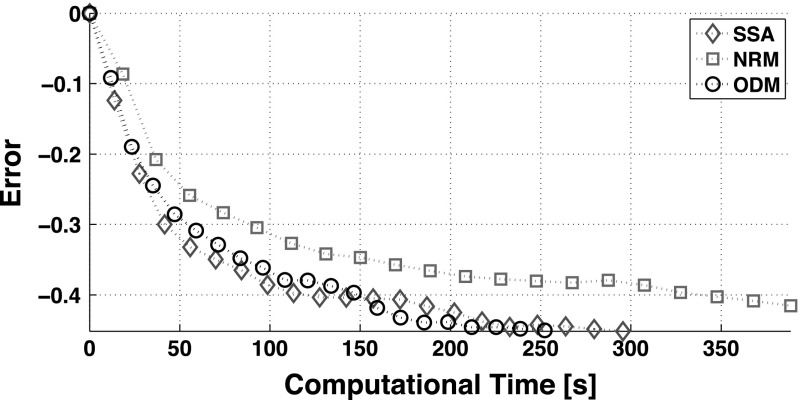
Approximation error of the simulation-based method in comparison to the exact analytical solution. The quantification error is defined by (38) and is measured every 50 runs of every simulation algorithm. The computational time required for the evaluation of the closed-form expressions is about $0.6~\mathrm s$. We choose $\eta = 50$ time-points along the transient response obtained by the simulation algorithms to evaluate the approximation error. The rest of the parameters values are as in Fig. 2

For this comparison, we opt not to include the hybrid method given that it might not be suitable for the study of this reaction network, as suggested by the results of Figs. 2, 3 and 4. For obtaining the marginal probability distributions $F_\mathrm{S}$, it is necessary to perform repeated runs of the simulation algorithms and average the results. In Fig. 5, we plot the error registered for every 50 runs of each simulation algorithm as a function of their computational time.

To conclude this section, we refer the interested reader to the SOM for the derivation of the steady-state, marginal probability density function via our analytical approach and a multiscale algorithm. In the following section, we analyse a ring of monomolecular reactions that models the ion gating driven by ryanodine receptors.

### Calcium Channel Mediated by Ryanodine Receptor

Cells are endowed with complex mechanisms to regulate the progression of messages encoded in calcium ions signals. Calcium ions pumps are one of the mechanisms with which calcium ions are released from the sarcoplasmic reticulum to the cytosol. A class of such pumps are the ryanodine receptors (RyR) and are known to mediate muscular function among many other cellular processes (Lanner et al. 2010).

Stern et al. (1999) modelled the RyR as a four state monomolecular reaction chain and Keener (2009) showed that the evolution of the time-dependent probability density functions associated to the gate activity belongs to a subspace of lower dimensions. We make use of our results to derive an exact equilibrium probability distribution of the ryanodine-mediated calcium ion gates to be in inactive, $S_1$, or open, $S_4$, states, thus reducing the system description exactly. This gate can be modelled by

$$\begin{aligned}&S_1 \mathop {\rightleftharpoons }\limits _{k_{\mathrm {b} 1}\kappa ^2}^{k_{\mathrm {f} 1}} S_2 \mathop {\rightleftharpoons }\limits _{k_{\mathrm {b} 2}}^{k_{\mathrm {f} 2}\kappa } S_3 \mathop {\rightleftharpoons }\limits _{k_{\mathrm {b} 3}}^{k_{\mathrm {f} 3}\kappa ^2} S_4,\\&S_4 \mathop {\rightleftharpoons }\limits _{k_{\mathrm {b} 4}}^{k_{\mathrm {f} 4}\kappa } S_1. \end{aligned}$$

Here $\kappa$ is the number of calcium ions and the states $S_{2,3}$ denote intermediate configurations in which the calcium ions gate is closed. We take from Stern et al. (1999) the following parameters values:

$$\begin{aligned} \{ k_{\mathrm {f} 1}, k_{\mathrm {f} 2}, k_{\mathrm {f} 3}, k_{\mathrm {f} 4} \}&= \{0.06,0.005,35\gamma ^2,0.5\gamma \} [\mathrm {ms}^{-1}], ~ \\ \{ k_{\mathrm {b} 1}, k_{\mathrm {b} 2}, k_{\mathrm {b} 3}, k_{\mathrm {b} 4} \}&= \{35\gamma ^2,0.5\gamma ,0.06,0.005\} [\mathrm {ms}^{-1}]. \end{aligned}$$

In the definition of the parameters, we have introduced the factor $\gamma = 1\times 10^{-3} /( {\mathcal {N}} {\mathcal {V}} )$, to transform the kinetic constants from the deterministic setting reported in Stern et al. (1999) to the stochastic formulation. ${\mathcal {N}} = 6.02214\times 10^{23} [\mathrm {molecules} / \mathrm {mol}]$ is the Avogadro constant and $\mathcal {V}$ is the volume of the region in which the reactions are taking place. Here, we assume a volume of $1\text { microlitre }[\mu ~\mathrm {l}]$.

Our computational analysis (data not shown) concluded that the eigenvalues of $\mathbf {A}$ in (32) are simple and real for $\kappa = \{ 10,30,50,70\}\times 10^{15}$ ions of calcium. Furthermore, we consider 100 gates. By assuming that all gates are initially in the inactive state $S_1$, the equilibrium point ${\bar{\mathbf {c}}}$ in (33) becomes

$$\begin{aligned} {\bar{\mathbf {\mathbf {c}}}}= \varrho \begin{pmatrix} 5.4690\times 10^{13}\kappa ^3(6.3225\times 10^{15}\kappa ^2 + 5.4408\times 10^{33}\kappa + 4.2595\times 10^{52})\\ 1.9057\times 10^{53}\kappa (1.1281\times 10^{13}\kappa ^2 + 9.7075\times 10^{30}\kappa + 7.5999\times 10^{49}) \\ 1.2949\times 10^{84}\kappa ^2 + 1.582\times 10^{124}\kappa + 1.2385\times 10^{143}\\ 2.5730\times 10^{31}\kappa ^2(8.0931\times 10^{15}\kappa ^2 + 6.9642\times 10^{33}\kappa + 5.4506\times 10^{52}) \end{pmatrix}, \end{aligned}$$

where $\varrho := [(7.02\times 10^{11}\kappa + 4.22\times 10^{29})(4.94\times 10^{15}\kappa ^4 + 4.25\times 10^{33}\kappa ^3 + 6.4\times 10^{52}\kappa ^2 + 2.64\times 10^{70}\kappa + 2.07\times 10^{89})]^{-1}$. The numerical values above are rounded to four decimals due to space limitations and to enhance readability. With these values and definitions, we now focus on the derivation of a probability density function for the states $S_1$ and $S_4$, assuming a constant number of calcium ions and until the gates reach their final configuration $( t \rightarrow \infty )$. To obtain the marginal probability distribution for states $S_1$ and $S_4$, we note that in Proposition 2

$$\begin{aligned} {\mathcal {M}} \left( (S_1,S_4)^T ,0,{\tilde{\mathbf{q}}}^j \right) = \delta _{\varvec{0}}\qquad \forall \, j \ne 1, \end{aligned}$$

as all the gates are in state $$S_1$$ at time $$ t = 0$$. Moreover, as there are no synthesis of gates $$\mathbf {b} = \varvec{0}$$, from (21b) and Proposition 2 we note

$$\begin{aligned} \varvec{\nu }( t ) = \varvec{0}\quad \forall \, t \quad \implies \quad {\mathcal {P}} \left( (S_1,S_4)^T, \varvec{\tilde{\nu }} ( t ) \right) = \delta _{\varvec{0}}. \end{aligned}$$

Now, from the ODE governing $\mathbf q^1( t )$ in (21a) and accounting for the definition of the equilibrium point in (33), we have

$$\begin{aligned} {\tilde{\mathbf{q}}}^1 (\infty ) = \varrho \begin{pmatrix} 5.4690\times 10^{13}\kappa ^3(6.3225\times 10^{15}\kappa ^2 + 5.4408\times 10^{33}\kappa + 4.2595\times 10^{52})\\ 2.5730\times 10^{31}\kappa ^2(8.0931\times 10^{15}\kappa ^2 + 6.9642\times 10^{33}\kappa + 5.4506\times 10^{52} \end{pmatrix} . \end{aligned}$$

Hence, the marginal probability distribution defined in Proposition 2 reduces to

$$\begin{aligned} \lim _{ t \rightarrow \infty }F\left( \left( S_1,S_4 \right) ^T , t \right) = \mathcal {M} \left( \left( S_1,S_4 \right) ^T,100,{\tilde{\mathbf{q}}}^1 (\infty ) \right) . \end{aligned}$$

**Fig. 6:**
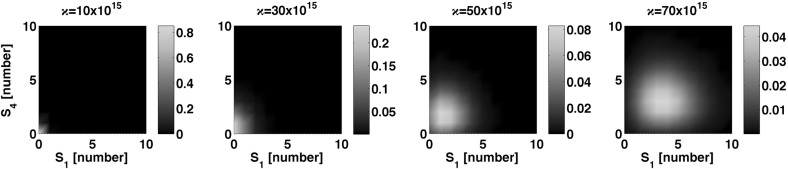
Marginal probability distribution for the inactive, $S_1$, and open, $S_4$, states of a ryanodine-mediated calcium ions gate. The number of surrounding calciums ions, $\kappa$, is assumed constant during the gate operation, yet different for each one of the panels above

It is important to note that ${\tilde{\mathbf{q}}}^1 (\infty )$ depends on the constant number of calcium ions $\kappa$. Figure 6 shows the the marginal probability distribution defined above as $\kappa$ varies. In Fig. 6 we note that as the number of calcium ions surrounding the gates increases, the number of open gates increases to expel the excess of calcium ions (Stern et al. 1999). For the volume considered, the concentration of calcium ions ranges from 15 to $120~[\mathrm {mM}]$, in contrast to the basal concentration of calcium (around $90[\mathrm {nM}]$) in diverse organisms such as *E. coli* (Gangola and Rosen 1987) and human nonexcitable cells (Korngreen et al. 1997).

Although one might expect that the number of open gates increases proportionally to the number of calcium ions, the nature of the gates is to remain open in a window of time for the exchange of ions, so as to regulate the calcium ions number within the sarco/endoplasmic reticulum and in the cytosol. After this exchange is finished, the gate remains closed (Stern et al. 1999). However, our calculations for the steady state suggest that when the surrounding calcium concentrations are orders of magnitude larger than the basal concentration of calcium, then some of the gates might remain open.

## Concluding Remarks

Chemical reactions are stochastic, discrete events. Especially when the number of molecules is low, this intrinsic randomness characterises the dynamical behaviour of the species’ molecular numbers. A usual approach to analyse these kind of systems is to use the SSA algorithm, namely, to generate a large number of trajectories of species’ molecular number and analyse them statistically. However, this approach might require a large computational load to yield accurate results.

In this paper we focused on exact solutions of the CME. By availing of an analytical solution, we derived closed-form expressions for the time-dependent probability associated with selected species of interest. In contrast to a numerical approach, these closed-form formulae can be reused for any initial condition as well as for exploring transient characteristics, such as maximum values of a probability of interest, time to approach equilibrium, steady-state probability distributions, among others. We note that in order to obtain similar results numerically, the CME would need to be solved (directly or approximated via the SSA) thousands of times, or even more, as these characteristics might be highly sensitive on initial conditions.

Our motivation to study only selected species is based on the limited availability of experimental data, hence the comparison between *in silico* and wet experiments is only possible for a reduced number of species. Moreover, in some pathways only some species or molecular configurations are relevant for downstream processes. Although we obtained time-dependent solutions of the CME for general reaction networks, we note that the number of states of such a equation increases rapidly as the number of molecules, reactions, and/or species increases; thereby, hindering an analytical treatment. In the case of monomolecular reaction networks, the ODEs required for obtaining the probability distribution of selected species are of the order of species involved, instead of the number of states of the CME.

In particular, we studied a protein autoactivation mechanism with nonlinear propensities. Also, we studied general and particular topologies of monomolecular reaction networks: an unbranched chain and rings of monomolecular reactions. For the former case, we compared the computational load of our results with that of simulation-based approaches. In such an example, we observed that the time required to implement our results require about the same time as some runs of the SSA, yet provide an exact description of the time-dependent, marginal probability density function for the species of interest. Additionally, we analysed a ring of reactions, which model calcium ion gates mediated by ryanodine receptors. There we provided an analytical solution for the equilibrium marginal probability distribution for the gates to be inactive or open as a function of the surrounding calcium in the sarco/endoplasmic reticulum.

By obtaining the analytical solution for those species of interest, we have provided the means to study systems that might become intractable by other methods. We foresee that these methods will assist on the understanding of processes underpinning selected phenomena in biochemistry, population dynamics, among other areas.

### Electronic supplementary material

Below is the link to the electronic supplementary material.

Supplementary material 1 (pdf 367 KB)

## Appendix

### Proof of Proposition 1

#### *Proof*

The solution of (10) is given by

39 $$\begin{aligned} \mathbf {y}( t )&={\mathcal {L}}^{-1} \left\{ \mathbf {C} \left( \zeta \mathbf {I - A} \right) ^{-1} \right\} \mathbf {e}_0\nonumber \\&={\mathcal {L}}^{-1} \left\{ \left( \det \left( \zeta \mathbf {I-A} \right) \right) ^{-1} \mathbf {M}(\zeta ) \right\} \mathbf {e}_0, \end{aligned}$$

where ${\mathcal {L}} ^{-1} \{\circ \}$ represents the inverse Laplace transform, $\zeta$ is the complex frequency variable, and $\mathbf {M}(\zeta )$ is as in (12a). The partial fraction expansion of the ratio of polynomials is given by Gantmakher (1959, Ch. V.3)

40 $$\begin{aligned} \frac{p(\zeta )}{q(\zeta )} = \sum _{\ell =1}^n \frac{p(\lambda _ \ell )}{\frac{\mathrm d}{\mathrm d \zeta } q\left( \lambda _{\ell } \right) } \frac{1}{\zeta -\lambda _{\ell }}, \end{aligned}$$

when the degree of $p(\zeta )$ is less than the degree of $q(\zeta )$ and $q(\zeta )$ has simple, real roots $\lambda _i$. In addition, the derivative of the determinant of a square, invertible matrix $\mathbf W(\zeta )$ is given by

41 $$\begin{aligned} \frac{\mathrm d}{\mathrm d \zeta } \det (\mathbf W(\zeta )) = \det (\mathbf W(\zeta )) \mathrm {trace}\left( \mathbf W^{-1}(\zeta ) \frac{\mathrm d}{\mathrm d \zeta } \mathbf {W}(\zeta ) \right) . \end{aligned}$$

Furthermore Horn and Johnson (2012)

42a $$\begin{aligned} \det \left( \mathbf {A}\right)&=\prod _{i=1}^{n} \lambda _i, \end{aligned}$$

42b $$\begin{aligned} \mathrm {trace}\left( \mathbf {A}^\alpha \right)&=\sum _{i=1}^{n} \lambda _i^{\alpha }. \end{aligned}$$

In turn, given that $\lambda _{\ell }$ are the eigenvalues of $\mathbf {A}$, the eigenvalues of $\zeta \mathbf {I-A}$ are $\zeta -\lambda _{\ell }$. Using expression (41) and (42), the derivative of $\det \left( \zeta \mathbf {I-A} \right)$ evaluated at $\zeta = \lambda _{\ell }$ yields

$$\begin{aligned} \left. \frac{\mathrm d}{\mathrm d \zeta }\det \left( \zeta \mathbf {I-A} \right) \right| _{\zeta =\lambda _{\ell }}&=\left. \left( \prod _{k=1}^n \zeta -\lambda _k \right) \left( \sum ^{n}_{j=1} \frac{1}{\zeta -\lambda _j} \right) \right| _{\zeta =\lambda _{\ell }},\nonumber \\&=\sum _{j=1}^{n} \prod _{k=1,k\ne j}^{n} \lambda _{\ell }-\lambda _k,\nonumber \\&=\prod _{k=1,k\ne \ell }^{n} \lambda _{\ell }-\lambda _k. \end{aligned}$$

By using the partial fraction expansion in (40) and the expression above, $\mathbf {y}( t )$ in (39) becomes

$$\begin{aligned} \mathbf {y}( t )&={\mathcal {L}}^{-1} \left\{ \sum _{\ell =1}^n \frac{\vartheta _{\ell }}{\zeta -\lambda _{\ell }} \mathbf {M}(\lambda _{\ell }) \right\} \mathbf {e}_0. \end{aligned}$$

The scalar $\vartheta _{\ell }$ was defined in (12b). By taking the inverse Laplace transform of the expression above we obtain (11), as desired. $\square$

### Proof of Proposition 4

#### *Proof*

From the definitions in (27), we note that $\mathrm {rank}\left( \mathbf {\mathbf {N_{\scriptscriptstyle {L}}}G} \right) = n-1$. Hence, form the Rank-Nullity theorem, the right null space of $\mathbf {N_{\scriptscriptstyle {L}}}\mathbf {G}$ is nontrivial with dimension 1. Let the columns of $\mathbf {R} \in {\mathbb {R}}^n$ span the right null space of $\mathbf {N_{\scriptscriptstyle {L}}}\mathbf {G}$, then

$$\begin{aligned} \mathbf {\mathbf {N_{\scriptscriptstyle {L}}}GR} = \varvec{0}. \end{aligned}$$

It is important to note that $\mathbf {\mathbf {N_{\scriptscriptstyle {L}}}G}$ is a tridiagonal matrix, hence a basis for its right null space can be computed recursively. For our case, this basis is given by

43 $$\begin{aligned} \mathbf R = \begin{pmatrix} \prod \limits _{i=1}^{n-1} \frac{k_{\mathrm {b} i}}{k_{\mathrm {f} i}}&\quad \prod \limits _{i = 2}^{n-1}\frac{k_{\mathrm {b} i}}{k_{\mathrm {f} i}}&\quad \ldots&\quad \frac{k_{\mathrm {b} n-1}}{k_{\mathrm {f} n-1}}&\quad 1 \end{pmatrix}^T. \end{aligned}$$

In turn, from (2), every fixed point ${\bar{\mathbf {\mathbf {c}}}}$ satisfies

$$\begin{aligned} \mathbf {\mathbf {N_{\scriptscriptstyle {L}}}G}{\bar{\mathbf {\mathbf {c}}}}= \varvec{0}. \end{aligned}$$

That is, we can express ${\bar{\mathbf {\mathbf {c}}}}$  as linear combination of the columns of $\mathbf R$:

44 $$\begin{aligned} \mathbf R \alpha = {\bar{\mathbf {\mathbf {c}}}}, \end{aligned}$$

for a suitable $\alpha \in {\mathbb {R}}$. The equation above is an algebraic equation with $n+1$ unknowns (one for each entry of ${\bar{\mathbf {\mathbf {c}}}}$ and another one for $\alpha$) and $n$ equations. To complete the algebraic system, we note that the left null space of $\mathbf {N_{\scriptscriptstyle {L}}}$ is nontrivial and of dimension 1. Let the rows of $\mathbf L$ span the left null space of $\mathbf {N_{\scriptscriptstyle {L}}}$. Hence, from (2), we have

$$\begin{aligned} \mathbf L \frac{\mathrm {d}}{\mathrm {d} t }\mathbf {c}( t ) = \varvec{0}. \end{aligned}$$

Integration of the equation above from 0 to $t$ yields

45 $$\begin{aligned} \mathbf L \mathbf {c}( t ) = \mathbf L \mathbf {c}_0, \quad \forall t \end{aligned}$$

In addition, for our case

46 $$\begin{aligned} \mathbf {L} = \varvec{1} ^T. \end{aligned}$$

From the equation above, equation (44) and since (45) is valid for any $t$  it follows

47 $$\begin{aligned} \mathbf L {\bar{\mathbf {\mathbf {c}}}}= \mathbf {L R}\alpha&=\mathbf {L \mathbf {c}_0}\nonumber \\ \alpha&=(\mathbf {LR} )^{-1} \mathbf L \mathbf {c}_0\\ \alpha&=\frac{\prod _{i=1}^{n-1} k_{\mathrm {f} i} }{\sum _{j=1}^{n} \left( \prod _{i=j}^{n-1} k_{\mathrm {b} i} \right) \left( \prod _{i=1}^{j-1} k_{\mathrm {f} i} \right) } \sum _{i=1}^{n}c_i(0).\nonumber \end{aligned}$$

For the last step in the derivation above, we used the definitions of $\mathbf R$ and $\mathbf L$ in (43) and (46), respectively. Finally, by substituting the definition of $\alpha$ above into the definition of the fixed point in (44), its $k$th coordinate has the form

$$\begin{aligned} \bar{c}_k&=\prod _{i=k}^{n-1} \frac{k_{\mathrm {b} i}}{k_{\mathrm {f} i}}\frac{\prod _{i=1}^{n-1} k_{\mathrm {f} i} }{\sum _{j=1}^{n} \left( \prod _{i=j}^{n-1} k_{\mathrm {b} i} \right) \left( \prod _{i=1}^{j-1} k_{\mathrm {f} i} \right) } \sum _{i=1}^{n}c_i(0). \end{aligned}$$

Further algebraic manipulation leads to the expression in (29). $\square$
