# Supplementary material for: Exact Probability Distributions of Selected Species in Stochastic Chemical Reaction Networks
Source: Bull Math Biol. 2014 Aug 26;76(9):2334–61. doi: 10.1007/s11538-014-9985-z (PMC4153981; doi:10.1007/s11538-014-9985-z)
Supplement: Supplementary file 1 — Supplementary material 1 (pdf 367 KB) [file 11538_2014_9985_MOESM1_ESM.pdf]

Supplementary Online Material to "Exact Probability Distributions of Selected Species in Stochastic Chemical Reaction Networks"

Fernando López-Caamal, Tatiana T. Marquez-Lago*
Integrative Systems Biology Unit, Okinawa Institute of Science and Technology,
Kunigami, Okinawa 904-0412, Japan

1 Solution of an ODE with Repeated Eigenvalues

Here we derive the closed form solution of a linear system of the form

$$\frac{\mathrm{d}}{\mathrm{d}t}\mathbf{e}(t) = \mathbf{A}\mathbf{e}(t), \quad \mathbf{e}(0) = \mathbf{e}_0, \tag{1a}$$

$$\mathbf{y}(t) = \mathbf{C}\mathbf{e}(t), \tag{1b}$$

where $\mathbf{e}(t) : \mathbb{R}_+ \to \mathbb{R}^n$, $\mathbf{A} \in \mathbb{R}^{n\times n}$, and $\mathbf{C}$ selects a number of states or linear combinations of $\mathbf{e}(t)$. We consider the case in which $\mathbf{A}$ has repeated eigenvalues.

To obtain the closed-form solution of (1), we express its transfer function as a finite sum of fractions. This transfer function is given by $\mathbf{C}\left(\zeta\mathbf{I} - \mathbf{A}\right)^{-1}$ and describes the relationship in the frequency domain between initial conditions $\mathbf{e}_0$ and output $\mathbf{y}(\zeta)$; that is to say

$$\mathbf{y}(\zeta) = \mathbf{C}\left(\zeta\mathbf{I} - \mathbf{A}\right)^{-1}\mathbf{e}_0. \tag{2}$$

Here $\mathbf{y}(\zeta)$ is the Laplace transform of $\mathbf{y}(t)$ and ζ represents the complex frequency variable that arises from the Laplace transform. The following proposition describes how to express such a transfer function as a finite sum of fractions.

Proposition 1. (Leyva-Ramos, 1991) *Consider a system of the form* (1)*, where the matrix* $\mathbf{A}$ *has* σ *eigenvalues with multiplicity* $\mu_\ell \in \mathbb{Z}\,\forall\,\ell \in [1,\sigma]$*. The partial fraction expansion of* $\mathbf{C}\left(\zeta\mathbf{I} - \mathbf{A}\right)^{-1}$ *is given by*

$$\mathbf{C}\left(\zeta\mathbf{I} - \mathbf{A}\right)^{-1} = \sum_{\ell=1}^{\sigma}\sum_{i=1}^{\mu_\ell}\frac{1}{(\zeta-\lambda_\ell)^i}\mathbf{K}_{\ell i}, \tag{3}$$

where

$$\begin{pmatrix}\mathbf{K}_{11}\\ \vdots \\ \mathbf{K}_{1\mu_1}\\ \vdots \\ \mathbf{K}_{\sigma\mu_\sigma}\end{pmatrix} = \left(\mathbf{V}^{-1}\otimes\mathbf{C}\right)\begin{pmatrix}\mathbf{I}_n\\ \mathbf{A}\\ \mathbf{A}^2\\ \vdots\\ \mathbf{A}^{n-1}\end{pmatrix}, \tag{4}$$

$\otimes$ *is the Kronecker product, and* $\mathbf{V}$ *is the Vandermonde matrix formed from the eigenvalues of* $\mathbf{A}$.

*Corresponding Author: Email tatiana.marquez@oist.jp

The Vandermonde Matrix of n numbers λ_ℓ, where λ_i is repeated μ_i times is (Leyva-Ramos, 1991)

$$\mathbf{V} := \begin{pmatrix} 1 & \dots & 1 & 0 & 0 & \dots & 0 & \dots & 1 \\ \lambda_1 & \dots & \lambda_i & 1 & 0 & \dots & \dots & \dots & \lambda_\sigma \\ \lambda_1^2 & \dots & \lambda_i^2 & 2\lambda_i & 1 & \dots & \dots & \dots & \lambda_\sigma^2 \\ \lambda_1^3 & \dots & \lambda_i^3 & 3\lambda_i^2 & 3\lambda_i & \dots & \dots & \dots & \lambda_\sigma^3 \\ \vdots & \vdots & \vdots & \vdots & \vdots & \vdots & \vdots & \vdots & \vdots \\ \lambda_1^{n-1} & \dots & \lambda_i^{n-1} & \binom{n-1}{1}\lambda_i^{n-2} & \binom{n-1}{2}\lambda_i^{n-3} & \dots & \binom{n-1}{\mu_i-1}\lambda_i^{n-\mu_i} & \dots & \lambda_\sigma^{n-1} \end{pmatrix}.$$

In the equation above, the superindex denotes exponentiation. By using the partial fraction expansion in (3) and by taking the inverse Laplace transform of (2), we obtain the solution of (1):

$$\mathbf{y}(t) = \sum_{\ell=1}^{\sigma} \exp(\lambda_\ell t) \sum_{i=1}^{\mu_\ell} \frac{t^{i-1}}{(i-1)!} \mathbf{K}_{\ell i} \mathbf{e}_0.$$

In Section 3.2 of the main manuscript, we are interested in deriving the solution of the following dynamical systems

$$\frac{\mathrm{d}}{\mathrm{d}t}\mathbf{q}^j(t) = \mathbf{A}\mathbf{q}^j(t), \qquad \mathbf{q}^j(0) = \boldsymbol{\varepsilon}^j, \quad \forall j \in [1, n], \tag{5a}$$
$$\tilde{\mathbf{q}} = \mathbf{C}\mathbf{q}$$
$$\frac{\mathrm{d}}{\mathrm{d}t}\boldsymbol{\nu}(t) = \mathbf{A}\boldsymbol{\nu}(t) + \mathbf{b}, \qquad \boldsymbol{\nu}(0) = \mathbf{0}. \tag{5b}$$
$$\tilde{\boldsymbol{\nu}} = \mathbf{C}\boldsymbol{\nu}$$

Here $\mathbf{C}$ is a matrix of appropriate dimensions that select the entries of interest of $\mathbf{q}^j$ and $\boldsymbol{\nu}$; and $\boldsymbol{\varepsilon}^j$ is the jth column of an identity matrix. When $\mathbf{A}$ has σ eigenvalues each with multiplicity μ_ℓ, we can avail of Proposition 1 of this SOM to obtain

$$\tilde{\mathbf{q}}^j(t) = \sum_{\ell=1}^{\sigma} \exp(\lambda_\ell t) \sum_{i=1}^{\mu_\ell} \frac{t^{i-1}}{(i-1)!} \mathbf{k}_{\ell i}^j, \tag{6a}$$
$$\tilde{\boldsymbol{\nu}}(t) = -\sum_{\ell=1}^{\sigma} \exp(\lambda_\ell t) \sum_{i=1}^{\mu_\ell} \frac{t^{i-1}}{(i-1)!} \mathbf{K}_{\ell i} \bar{\boldsymbol{\nu}} + \mathbf{C}\bar{\boldsymbol{\nu}}(t), \tag{6b}$$

where $\mathbf{K}_{\ell i}$ is given in (4), $\mathbf{k}_{\ell i}^j$ is the jth column of $\mathbf{K}_{\ell i}$, and $\bar{\boldsymbol{\nu}}$ represents the steady state of (5b).

2 Steady-State Marginal Probability Distribution for an Unbranched Monomolecular Reaction Chain

This section is a follow-up of the case-study in Section 4.2 of the main manuscript. The reaction network studied is

$$S_1 \underset{k_{\mathrm{b}1}}{\overset{k_{\mathrm{f}1}}{\rightleftharpoons}} S_2 \underset{k_{\mathrm{b}2}}{\overset{k_{\mathrm{f}2}}{\rightleftharpoons}} \dots \underset{k_{\mathrm{b}n-1}}{\overset{k_{\mathrm{f}n-1}}{\rightleftharpoons}} S_n, \tag{7a}$$
$$S_i \xrightarrow{k_{\mathrm{d}i}} 0, \tag{7b}$$
$$0 \xrightarrow{k_{\mathrm{s}i}} S_i. \tag{7c}$$

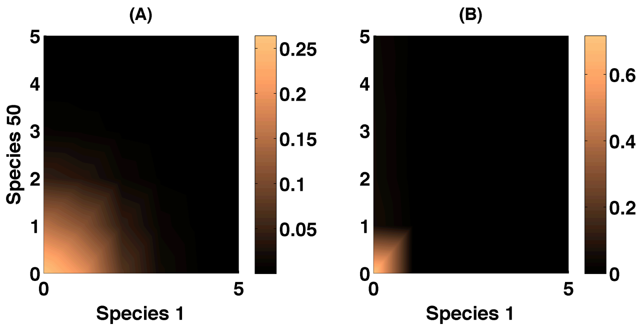

Figure 1: *Steady-States obtained via **(A)** our analytical approach and **(B)** the multiscale methodology in (Cotter et al., 2011). The parameters are those of Figure 3 of the main manuscript.*

Here, we derive steady state distribution by two different methods: (i) the analytical expressions in the (23) and Proposition 2 of the main manuscript and (ii) the multiscale methodology by Cotter et al. (2011), that assumes that the evolution of the slow species is well approximated by a Langevine process.

For our analytical approach, we note that the steady-state probability density function described in Proposition 2 of the main manuscript is given by the expression

$$\lim_{t\to\infty} F(\mathbf{y},t) = \mathcal{P}\left(\mathbf{y}, \tilde{\boldsymbol{\nu}}(\infty)\right) * \mathcal{M}\left(\mathbf{y}, \xi_1, \tilde{\mathbf{q}}^1(\infty)\right) * \ldots * \mathcal{M}\left(\mathbf{y}, \xi_n, \tilde{\mathbf{q}}^n(\infty)\right),$$

where $\tilde{\mathbf{q}}^j(\infty)$ and $\tilde{\boldsymbol{\nu}}(\infty)$ is an abuse of notation, which we use to denote the steady state of the ODEs in (5). In turn, $\mathbf{y}$ gathers the molecular species number of the first and last species, i.e. $\mathbf{y} := (s_1 \quad s_n)^T$. When all the kinetic parameters of (7) are positive, the eigenvalues of the matrix $\mathbf{A}$ in (5) are all negative. Hence from (23) of the main manuscript, we conclude that

$$\tilde{\mathbf{q}}^j(\infty) = \mathbf{0},$$
$$\tilde{\boldsymbol{\nu}}(\infty) = -\mathbf{C}\mathbf{A}^{-1}\mathbf{b}.$$

The matrix $\mathbf{C}$ is designed to obtain the first and last entry of the vector $\mathbf{A}^{-1}\mathbf{b}$. By using these expressions in the steady-state distribution above, we obtain

$$\lim_{t\to\infty} F(\mathbf{y},t) = \mathcal{P}\left(\mathbf{y}, -\mathbf{C}\mathbf{A}^{-1}\mathbf{b}\right).$$

We note that this is the exact solution for the steady state of the chemical master equation for the selected species.

Now, the multiscale methodology in (Cotter et al., 2011) requires the identification of slow and fast varying species. Once this separation of scales is identified, the probability distributions that characterises the behaviour of the fast variables is obtained via stochastic simulations for each possible combination of values of the slow variables. Please, refer to (Cotter et al., 2011, Sec. III) for a detailed explanation. The outcome of these simulations is used to approximate the steady-state of the slow species via the steady-state of a Fokker-Planck equation. To obtain the solution of such a Fokker-Planck equation, we availed of the Finite Volume Method described in (Sjöberg et al., 2009, Sec. 2.2). Figure 1 depicts the comparison of the outcome of both approaches.

Finally, we compared the computational time required to obtain such a steady-state distribution by means of the expression

$$r = \log_{10}\left(\frac{t_{\mathrm{MS}} - t_{\mathrm{A}}}{t_{\mathrm{A}}}\right). \tag{8}$$

Here t_{MS} denotes the computational time required by the method in (Cotter et al., 2011) and t_{A} represents the time required by the analytical method. Figure 2 depicts such a computational time comparison for different lengths n of the reaction network (7).

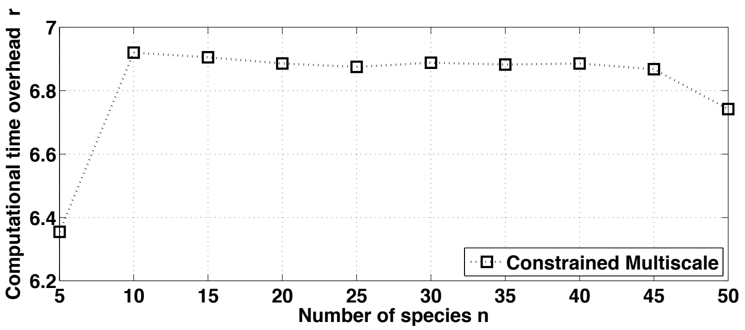

Figure 2: *Computation time overhead of the multiscale algorithm with respect to the analytical approach. The variable r has been defined in* (8)*.*

References

Cotter, S. L., K. C. Zygalakis, I. G. Kevrekidis, and R. Erban (2011). A constrained approach to multiscale stochastic simulation of chemically reacting systems. *The Journal of chemical physics 135*(9), 094102.

Leyva-Ramos, J. (1991). A method for partial-fraction expansion of transfer matrices. *Automatic Control, IEEE Transactions on 36*(12), 1472–1475.

Sjöberg, P., P. Lötstedt, and J. Elf (2009). Fokker–planck approximation of the master equation in molecular biology. *Computing and Visualization in Science 12*(1), 37–50.
